# Supplementary material for: Single-neuron correlates of visual consciousness in human lateral occipital complex
Source: Nat Commun. 2025 Dec 15;16:11008. doi: 10.1038/s41467-025-67077-w (PMC12705761; doi:10.1038/s41467-025-67077-w)
Supplement: Supplementary file 1 — Supplementary Information [file 41467_2025_67077_MOESM1_ESM.pdf]

Supplementary tables and figures

		Array 1	Array 2	Array 3	Array 4	Array 5
Scrambled versus non-scrambled	Visually responsive	51	92	56	35	24
	Selective (p<0.05)	12	46	47	31	19
	Selective (p<0.01)	6	39	45	21	16
	Selective (p<0.001)	4	34	41	15	15
	Nsc > Scr (p<0.001)	4	28	41	15	15
	Scr > Nsc (p<0.001)	0	6	0	0	0
	Average d’	0,26	1,08	2,70	2,53	1,79
Category selectivity	Visually responsive	52	92	72	66	32
	Selective (ANOVA p<0.05)	5	14	15	56	13
	Body selective (p<0.05)	3	4	5	51	4
	Face selective (p<0.05)	2	8	8	5	4
	Face and body selective (p<0.05)	0	0	0	0	5
	Average d’ (body vs face)	0.67	-0.57	0.22	1.93	0.49
	Average d’ (body vs object)	0.46	-0.60	0.06	2.29	1.06
	Average d’ (face vs object)	-0.33	0.77	-0.11	0.15	1.14

Supplementary Table S1. Number of visually responsive and selective MUA for the LO localizer (scrambled versus non-scrambled – Scr vs Nsc), and the category selectivity experiment. For both experiments, responsiveness is assessed using a two-sided dependent samples t-test comparing response window to baseline ($p < 0.005$). A two-sided independent samples t-test was used to assess selectiveness for scrambled vs non-scrambled images ($p < 0.05$). One-way ANOVA was used to assess selectiveness for category selectivity (between any category in general, $p < 0.05$), and Tukey’s HSD was used to test for selectivity between individual categories ($p < 0.05$).

	Mondrian	Human face	Human Body	Objects/fruits	Total
Array 2	91	55	44	30	92
Array 3	5	1	9	4	12
Array 4	1	6	16	1	22
Array 5	1	2	2	0	4

Supplementary Table S2. Number of responsive MUAs to selected categories after a 16 ms stimulus presentation, and to the masking Mondrian during a 250 ms stimulus presentation. The last column (Total) represents the total number of visually responsive multi-units. Note that some multi-units respond to multiple categories, so the total is not simply the sum of the visually responsive channels for other categories.

Array	Target stimulus	Preferred class	Response window (ms)	Amount selective SUA	<= 66 ms	83-132 ms	>= 150 ms
Array 2	Categorical	Face	130 - 280	3	0.018	0.122	6.28×10^{-4}
	Non-Scrambles vs scrambled	Non-scrambled	130 - 280	8	0.186	1.53×10^{-5}	3.94×10^{-4}
Array 4	Categorical	Body	200 - 350	6	0.824	2.66×10^{-4}	0.556

Supplementary Table S3. Backward masking. Significance levels (two-sided independent samples t-test) for the z-normalized net spike rate for all selective single-units (perceived preferred class versus non-perceived preferred class) during backward masking within the response window.

		Interval (ms)	Signal Loss 16 - 183 ms %	Signal Loss 16 - 183 ms p	Signal Loss 16 - 66 ms %	Signal Loss 16 - 66 ms p	Signal Loss 83 - 132 ms %	Signal Loss 83 - 132 ms p	Signal Loss 150 - 183 ms %	Signal Loss 150 - 183 ms p	First significant bin (ms)	Analyzed class
Array 1 – Category	Responsive	60 - 210	-8.9	0.349	0.4	0.965	*NA*	*NA*	*NA*	*NA*	/	Face
Array 2 - Category	Responsive	80 - 230	26.6	$2.01 * 10^{-6}$	22.1	0.007	25.9	0.005	73.8	$1.23 * 10^{-10}$	105	Face
	Selective		59.4	$2.71*10^{-11}$	53.8	0.005	52.1	0.002	56.8	$2.94 *10^{-4}$		
Array 2 - Scrambled vs Non-scrambled	Responsive	80 - 230	15.4	$5.62 * 10^{-13}$	11.5	0.033	14.9	$1.01 * 10^{-5}$	33.8	$1.41 * 10^{-12}$	95	Non - scrambled
	Selective		39.4	$1.24*10^{-32}$	43.5	$2.48 *10^{-7}$	26.3	$1.09 *10^{-5}$	27.1	$2.3 * 10^{-2}$		
Array 4 - Category	Responsive	200 - 350	44.6	$3.52 * 10^{-11}$	0.8	0.978	43.4	$3.32 * 10^{-7}$	29.6	0.0166	165	Body
	Selective		42.4	$1.15 * 10^{-8}$	9.6	0.789	39.9	$1.48* 10^{-4}$	9.8	0.470		
Array 5 - Category	Responsive	140 - 290	28.3	0.191	-18.9	0.483	9.83	0.82	36.9	0.246	/	Face

Supplementary Table S4. Signal loss for unperceived stimuli during backward masking. This table summarizes the percentage signal loss ('%') and p-values ('p'; two-sided independent samples t-test) for unperceived stimuli compared to perceived stimuli during backward masking. A positive percentage indicates that perceived stimuli elicited a larger response than unperceived stimuli within the specified 150 ms interval (see Methods). The "First significant bin" column identifies the earliest of three consecutive time bins (in ms) where the response to perceived stimuli was significantly greater than for unperceived stimuli ($p < 0.05$, one-sided permutation test), calculated across all delays (16–183 ms). Values marked as "NA" indicate instances where no unperceived stimuli were recorded because the patient responded correctly 100% of the time. Arrays 1 and 5 do not report a first significant bin because no three consecutive bins met the criterion of perceived > unperceived ($p < 0.05$).

		-300 ms till 0 ms				100 ms till 400 ms			
	Delay Group (ms)	Decoder		Spike rate		Decoder		Spike rate	
		AUC	p	Δ	p	AUC	p	Δ	p
Array 1 – Category	16 – 183	0.507	0.341	0.01	0.155	0.495	0.959	0.05	0.001
	16 – 66	0.544	1.28×10^{-7}	0.02	0.114	0.568	8.58×10^{-21}	0.01	0.317
	83 – 132	0.541	2.17×10^{-8}	0.03	0.172	0.544	0.005	0.07	0.066
	150 – 183	0.470	0.365	-0.02	0.816	0.566	1.75×10^{-4}	0.14	1.01×10^{-4}
Array 2 – Category	16 – 183	0.499	1.000	0.00	0.318	0.646	3.93×10^{-25}	-0.01	0.074
	16 – 66	0.621	1.43×10^{-31}	-0.02	0.848	0.475	0.999	-0.02	0.832
	83 – 132	0.600	1.12×10^{-44}	0.03	0.018	0.721	4.72×10^{-100}	0.04	0.001
	150 – 183	0.688	5.80×10^{-77}	0.01	0.328	0.669	1.48×10^{-60}	0.06	2.53×10^{-4}
Array 2 – Scrambled vs Non-scrambled	16 – 183	0.501	0.499	0.04	4.6×10^{-7}	0.769	0.496	0.07	6.7×10^{-17}
	16 – 66	0.499	0.423	0.03	0.059	0.593	7.96×10^{-8}	0.05	0.025
	83 – 132	0.533	3.64×10^{-5}	0.05	1.63×10^{-5}	0.728	4.76×10^{-98}	0.03	0.013
	150 – 183	0.581	1.17×10^{-13}	-0.03	0.978	0.627	7.56×10^{-24}	0.06	5.4×10^{-4}
Array 4 – Category	16 – 183	0.500	1.000	-0.04	0.999	0.616	3.52×10^{-32}	0.16	$3.52 * 10^{-32}$
	16 – 66	0.488	0.999	-0.02	0.894	0.531	1.09×10^{-5}	0.04	0.038
	83 – 132	0.512	0.0072	-0.08	1.000	0.528	3.34×10^{-9}	0.15	4.39×10^{-10}
	150 – 183	0.485	1.000	-0.03	0.911	0.678	1.93×10^{-82}	0.19	8.13×10^{-12}
Array 5 – Category	16 – 183	0.500	1.000	-0.06	0.999	0.528	1.5×10^{-8}	-0.03	0.824
	16 – 66	0.489	1.000	-0.02	0.693	0.552	3.4×10^{-23}	-0.06	0.954
	83 – 132	0.491	0.953	0.01	0.409	0.495	0.827	-0.02	0.628
	150 – 183	0.468	1.000	-0.08	0.987	0.479	0.999	0.01	0.428

Supplementary Table S5. Backward masking: exclusion of baseline differences between perceived and non-perceived target stimuli using a logistic regression decoder and raw spike rates. Decoder results (AUC) from logistic regression trained on the z-normalized raw spike rate - without baseline subtraction - to differentiate whether a target stimulus is perceived, based upon baseline activity (-300 till 0 ms). P-value is the result of a one-sided dependent samples t-test of AUC results versus AUC results from randomly shuffled labels (250 times). As control, same type of decoder was trained on the interval 100-400 ms after stimulus onset. Δ spike rate is the z-normalized raw spike rate difference between perceived and non-perceived target stimuli. P-value is the result of a one-sided independent samples t-test comparing the z-normalized raw spike rate responses during perceived and non-perceived target stimuli. Baseline changes are significant if $p<0.05$.

	A1 - Category	A2 - Non-Scrambled vs Scrambled		A4 - Category		A5 - Category	
Delay (ms)	Responsive	Responsive	Selective	Responsive	Selective	Responsive	Selective
16	0.682	**0.016**	0.120	**0.013**	0.077	**0.040**	0.323
32	0.209	0.159	0.138	**0.022**	0.199	**0.004**	0.157
50	0.849	0.171	0.226	0.207	0.105	**0.027**	0.113
66	NaN	0.559	0.100	**0.009**	0.138	0.546	1.000
83	0.058	**0.002**	0.2070	0.122	0.362	**0.005**	0.067
100	0.145	**0.003**	0.355	**0.006**	0.071	0.088	1.000
116	0.161	**$3.0 * 10^{-5}$**	0.071	0.145	**0.009**	0.395	1.000
132	**0.003**	**$<10^{-6}$**	0.335	0.455	0.727	0.245	1.000
150	**0.034**	**$<10^{-6}$**	**0.031**	0.203	0.241	0.050	0.512
166	**0.001**	**$<10^{-6}$**	**0.015**	0.213	0.426	0.094	1.000
183	**0.004**	**$<10^{-6}$**	0.482	0.77	0.184	0.088	1.000
200	**0.001**	NaN	NaN	NaN	NaN	0.096	0.314
Number of SUA	42	79	8	17	6	10	1

Supplementary Table S6. Backward masking. P-values (one-sided Silverman's test) for bimodal SUA response patterns during backward masking. The neural response is bimodal when $p < 0.05$. Calculations were based on the class eliciting the strongest response (Figure S2 and S4): 'face' for A1 and A5, 'body' for A4 and 'non-scrambled' images for A2. Notably, a bimodal response pattern was observed in visually responsive units for A1 and A2 at delays ≥ 132 ms and ≥ 83 ms, respectively. In contrast, A4 and A5, as well as the selective units from A2 (which are theoretically less responsive to the Mondrians), did not exhibit bimodal responses for longer delays. Some bimodality was observed in responsive units at shorter delays in A4 and A5, likely due to Type 1 errors (see Methods for peak identification criteria and an explanation why low firing rates increase the risk of false second-peak identification and thereby Type 1 errors; see also Figure S8, S9 and S29). 'NaN' values indicate non-recorded conditions. No selective units were identified in A1.

		Array 2	Array 3	Array 4	Array 5
MUA	Visually responsive	81	28	13	20
	Face responsive	77	24	12	18
	Place responsive	67	18	1	10
	Face selective	36	6	8	10
	Place selective	3	7	0	0
SUA	Visually responsive	39	12	4	6
	Face responsive	37	11	4	5
	Place responsive	22	8	0	5
	Face selective	24	4	4	5
	Place selective	4	3	0	0
	Sustained responders	3	1	2	0
	Inhibitory responders	10	2	1	1
	Sustained + inhibitory	12	2	2	1

Supplementary Table S7. Flash suppression. Number of visually responsive and stimulus selective multi- and single units. Responsiveness is assessed using a two-sided dependent samples t-test comparing response window to baseline ($p < 0.005$). A two-sided independent samples t-test was used to assess selectiveness ($p < 0.05$).

		Array 1	Array 2	Array 3	Array 4	Array 5
MUA	Visually responsive	32	74	32	12	21
	Face selective	22	35	8	4	0
	Place selective	0	4	4	0	8
SUA	Visually responsive	17	28	14	4	15
	Face selective	8	16	5	4	2
	Place selective	0	6	1	0	5

Supplementary Table S8. Binocular rivalry. Number of visually responsive and stimulus selective multi- and single units.

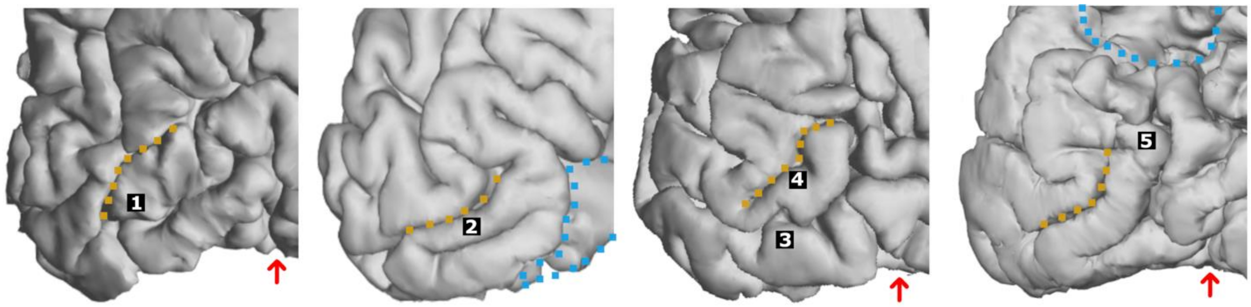

Supplementary figure S1. Anatomical locations of Utah arrays projected on 3D cortical renderings for all five arrays (4 subjects). MNI coordinates (X, Y, Z) are as follows: A1 (42, -76, -10), A2 (-35, -89, -8), A3 (-41, -83, 9), A4 (-38, -84, -5) and A5 (51, -66,19). Previous cavities are indicated by blue dotted lines. The brown dotted lines represent the lateral occipital sulcus. The red vertical arrows depict the pre-occipital notch.

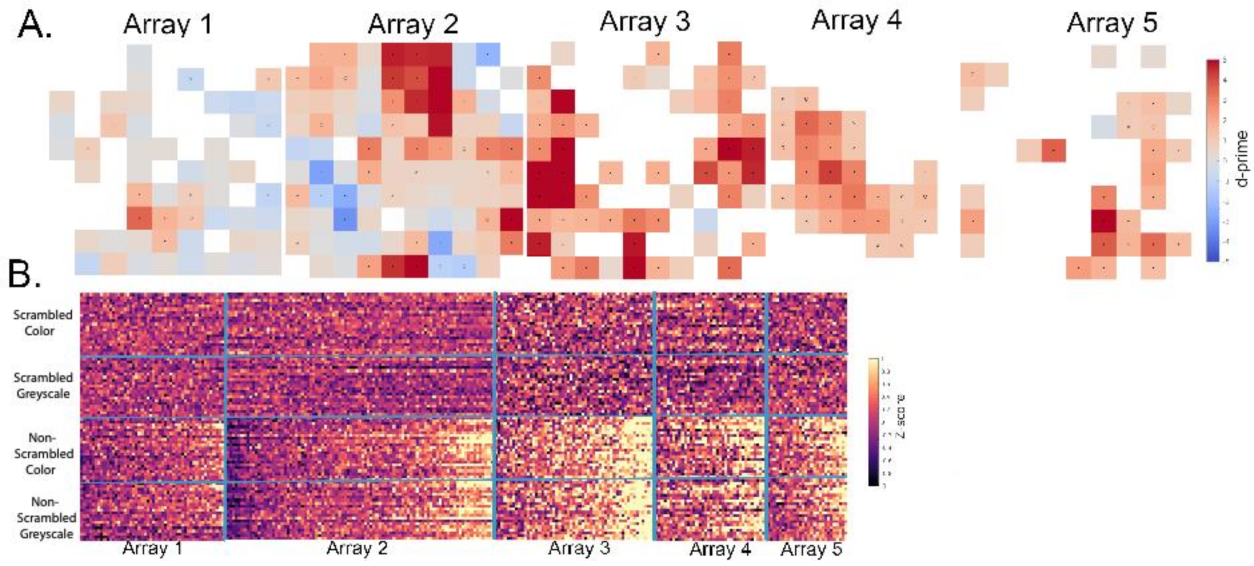

Supplementary figure S2. Effect of image scrambling on MUA. A) Two-dimensional representation of d' values across arrays, including only visually responsive multi-units. Significance of d-prime values is indicated within each square (two-sided permutation test: dot $p < 0.001$; circle $p < 0.01$; cross $p < 0.05$). **B)** Z-scored net MUA responses within the response window from all arrays for each individual stimulus. On the y-axis, each dot is grouped by category and represents a different stimulus; each dot on the x-axis represents a visually responsive MUA. The average net response to the scrambled category was subtracted from each stimulus for z-scoring, resulting in a mean Z-score of zero for the scrambled category. Within each array, MUA is sorted according to d' (Non-Scrambled vs Scrambled).

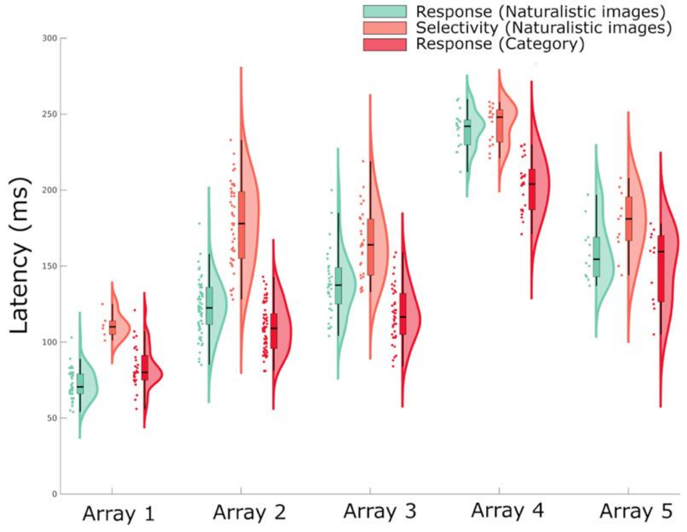

Supplementary figure S3. Response and selectivity latencies for naturalistic images and category stimuli per array. Combined scatter-, box- and violin plot showing response latency to naturalistic images (green) (non-scrambled condition from the LO localizer experiment) and to the category selectivity stimuli (red). Box plots indicate median (middle line), 25th, 75th percentile (box) and box limits $\pm$ 1.5 x interquartile range (whiskers). Orange plots indicate latency for emergence of selectivity between non-scrambled and scrambled images. The used intervals for response latencies for array 1 until 5 were respectively: [60 210], [80 230], [130 280], [200 350] and [130 290]. The used intervals for selectivity latencies for array 1 until 5 were respectively: [100 250], [130 280], [130 280], [200 350] and [140 290]. For array 4, response latency was markedly different for the naturalistic images (255 ms) compared to the category selectivity stimuli (205 ms). Since the categorical (body) stimuli were more optimal stimuli to drive neural responses in array 4, we opted to use [200 350] instead of [250 400] as the latency intervals.

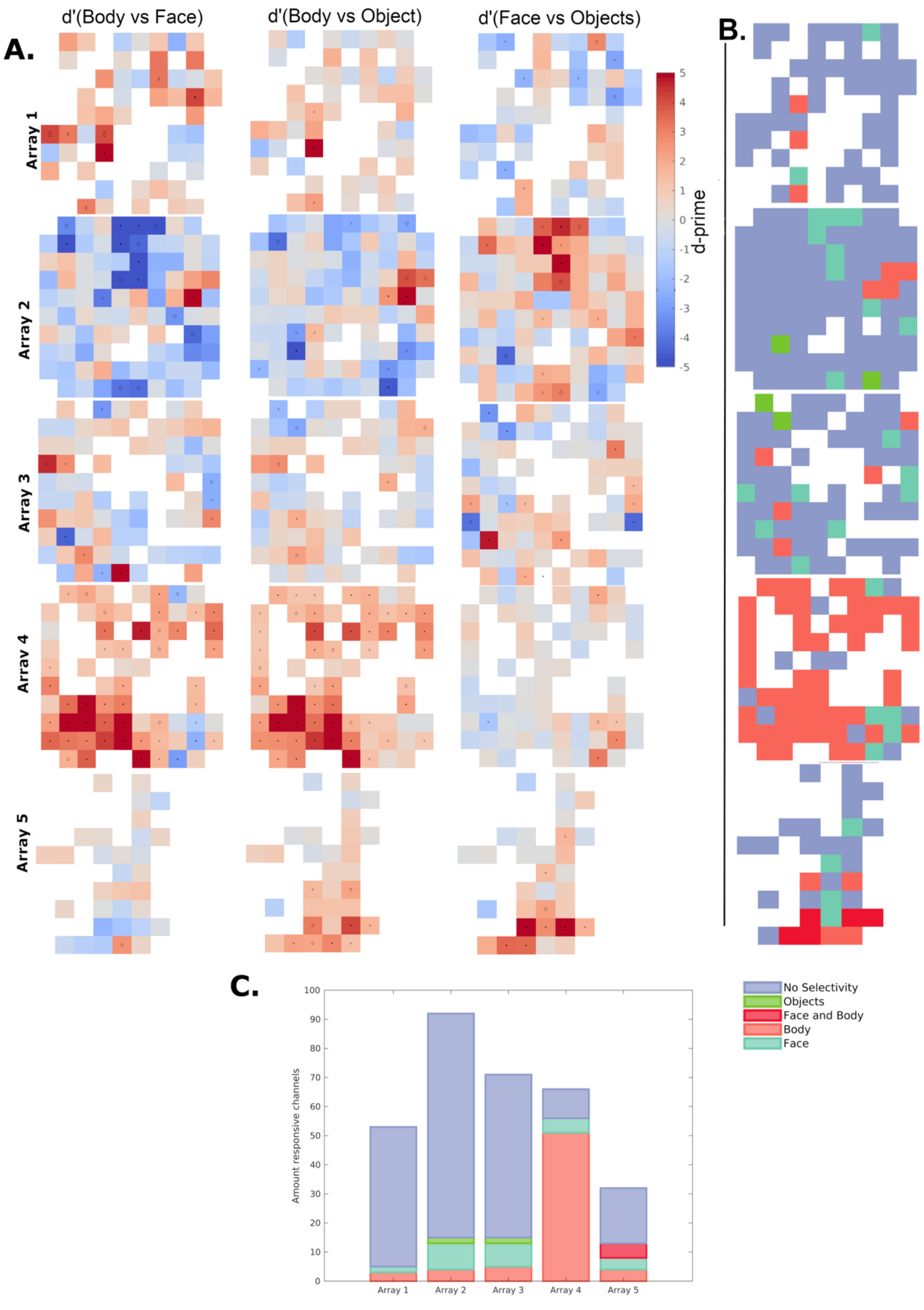
A.
d'(Body vs Face)
d'(Body vs Object)
d'(Face vs Objects)
Array 1
Array 2
Array 3
Array 4
Array 5
d-prime
5
4
3
2
1
0
-1
-2
-3
-4
-5
B.
C.
Amount responsive channels
100
90
80
70
60
50
40
30
20
10
0
Array 1
Array 2
Array 3
Array 4
Array 5
No Selectivity
Objects
Face and Body
Body
Face

Supplementary figure S4. Category Selectivity across arrays. A. Two-dimensional representation of d' values from visually responsive multi-units across arrays. Significance of d-prime values is indicated within each square (Tukey's HSD: dot $p < 0.001$; circle $p < 0.01$; cross $p < 0.05$). **B.** Two-dimensional visualization across each array of face, body, and object selectivity (see Methods). Note the strong body selectivity in array 4 as well as the strong face and body preference in array 5. **C.** Bar plot showcasing the number of visually responsive and category-selective channels per array. The same color coding applies to panels B and C.

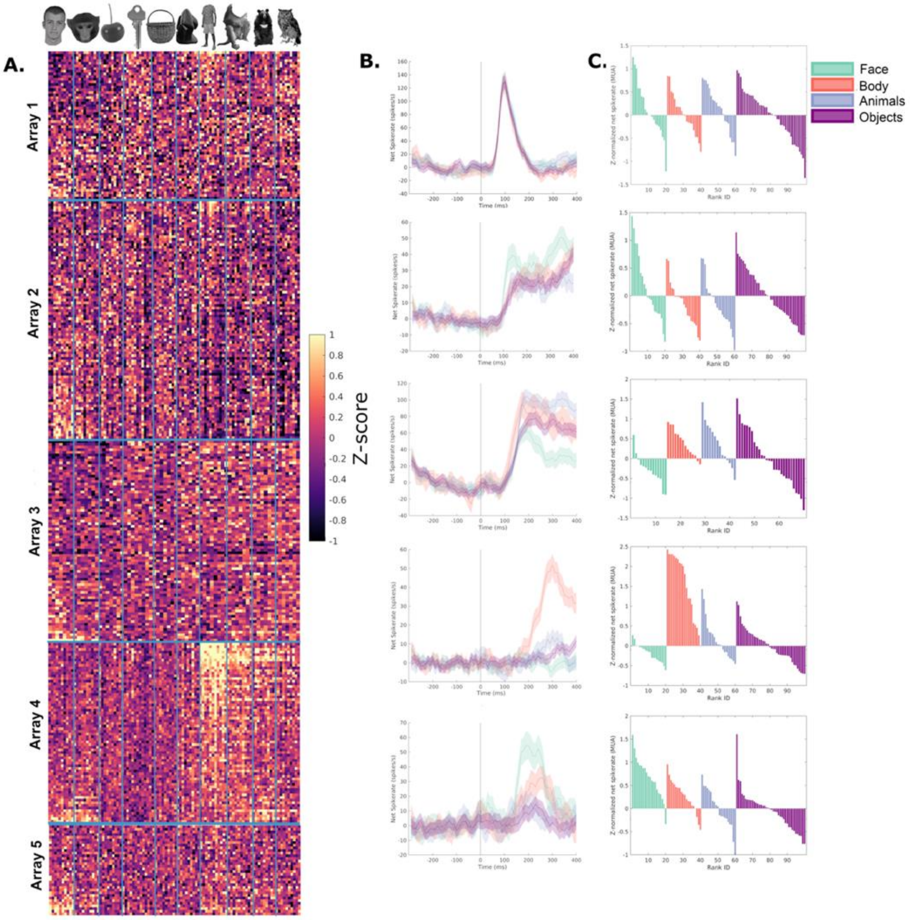

Supplementary figure S5. Category selectivity for individual MUA to individual stimuli. A. Z-scored net MUA responses per arrays for all individual stimuli. Stimuli were published in Popivanov, I. D., Jastorff, J., Vanduffel, W. & Vogels, R. Stimulus representations in body-selective regions of the macaque cortex assessed with event-related fMRI. Neuroimage 63, 723–741 (2012), Copyright Elsevier. Each dot on the x-axis is grouped per category and represents a different stimulus; each dot on the y-axis represents a visually responsive MUA. Average net response to the objects category was subtracted from each stimulus for Z-scoring, resulting in mean Z-score of zero for objects. Within each array, MUA was sorted according to d'(Face vs Body). **B and C.** Responses of a representative multi-unit example per array. **B.** Mean net spike rate per category for an example MUA per array. Shading represents standard error (N = number of trials per condition). **C.** Average net z-scored responses to individual stimuli ranked within their respective category (faces (human and monkey), bodies (human and monkey), animals (mammals and birds) and objects (objects-monkey, objects-human, fruits, sculptures)).

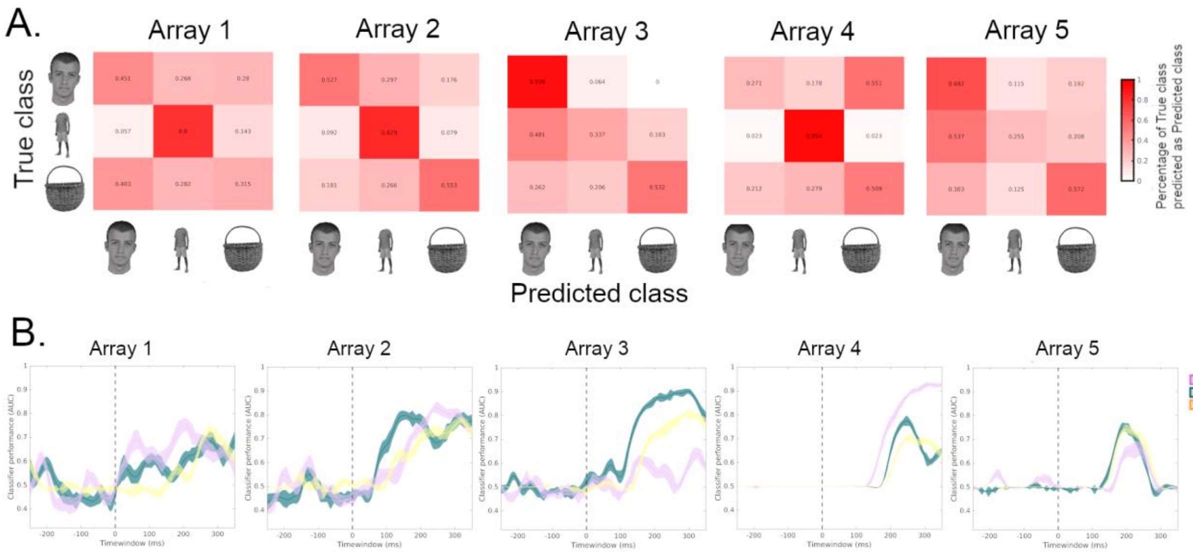

Supplementary figure S6. Classifier performance for individual categories. A) Confusion matrices per array at peak classification between human faces, human bodies and objects (objects-human, objects-monkey, fruits and sculptures). Stimuli were published in Popivanov, I. D., Jastorff, J., Vanduffel, W. & Vogels, R. Stimulus representations in body-selective regions of the macaque cortex assessed with event-related fMRI. Neuroimage 63, 723–741 (2012), Copyright Elsevier. Peak classification was obtained in interval [160-260], [180-280], [250-350], [260-360] and [140-240] for A1 to A5 respectively. Ground truth labels are presented on the Y-axis, actual predictions on the X-axis. The matrices are normalized across rows to highlight the proportion of true positives across each category. **B)** Average classification performance (AUC) over time for individual categories vs all other categories. Shading represents standard error classification AUC from 10 decoding repetitions. Vertical dotted lines mark stimulus onset. Times on the x-axis represent the middle of the 100 ms interval used for classification. Peak AUCs for the preferred class were 81% for A1 (face), 85% for A2 (body), 93% for A3 (face), 94% for A4 (body), and 75% for A5 (object).

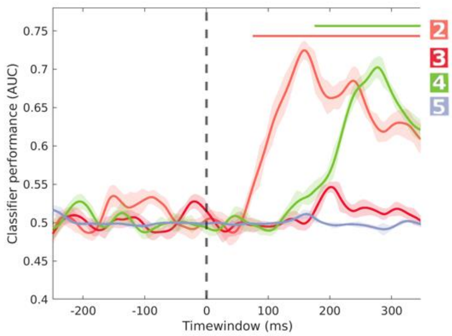

Supplementary Figure S7. Category selectivity: decoder performance for 16 ms stimulus presentations. Lines and shading represent the mean ± standard error classification AUC from 10 decoding repetitions. For each array, the best class was decoded from other classes (human face, human body or objects). This control experiment was not performed for array 1. Horizontal bars indicate significance compared to random label shuffling ($p < 0.01$; one-sided permutation test), and are shown only when at least five consecutive bins were significantly different. Colored numbers refer to the array numbers.

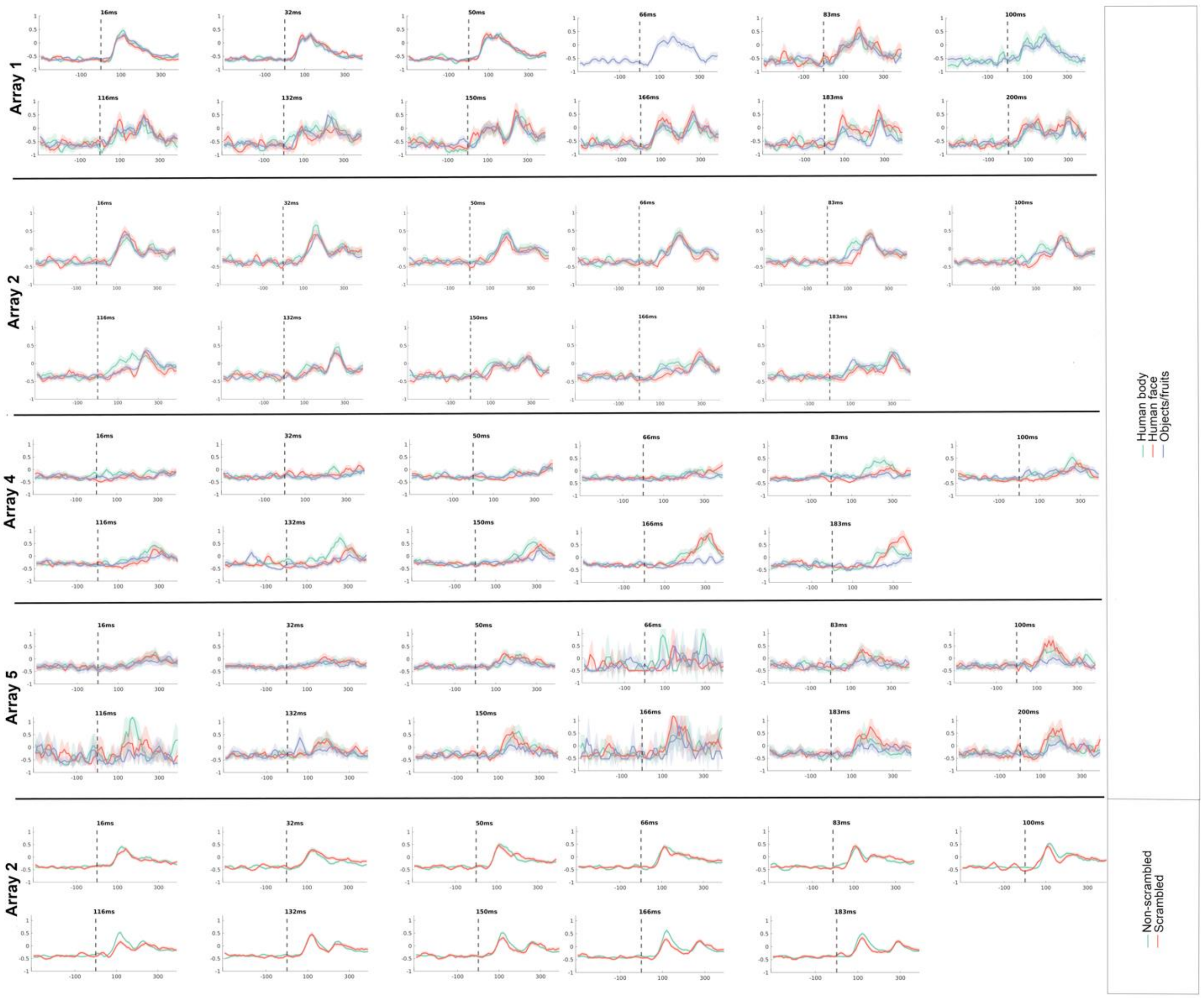

Supplementary Figure S8. Backward masking: average Z-normalized response per delay for all <u>visually responsive</u> single units. No distinction is made between perceived and non-perceived target stimuli. In each plot, the x-axis represents time (in milliseconds), and the y-axis represents the average Z-normalized net response for all visually responsive single units. The dotted line indicates the onset of the target stimulus. Note the distinct responses for both the target stimuli and Mondrian in arrays 1 and 2 for longer delays. Shading represents standard error (N = number of trials x number of visually responsive channels).

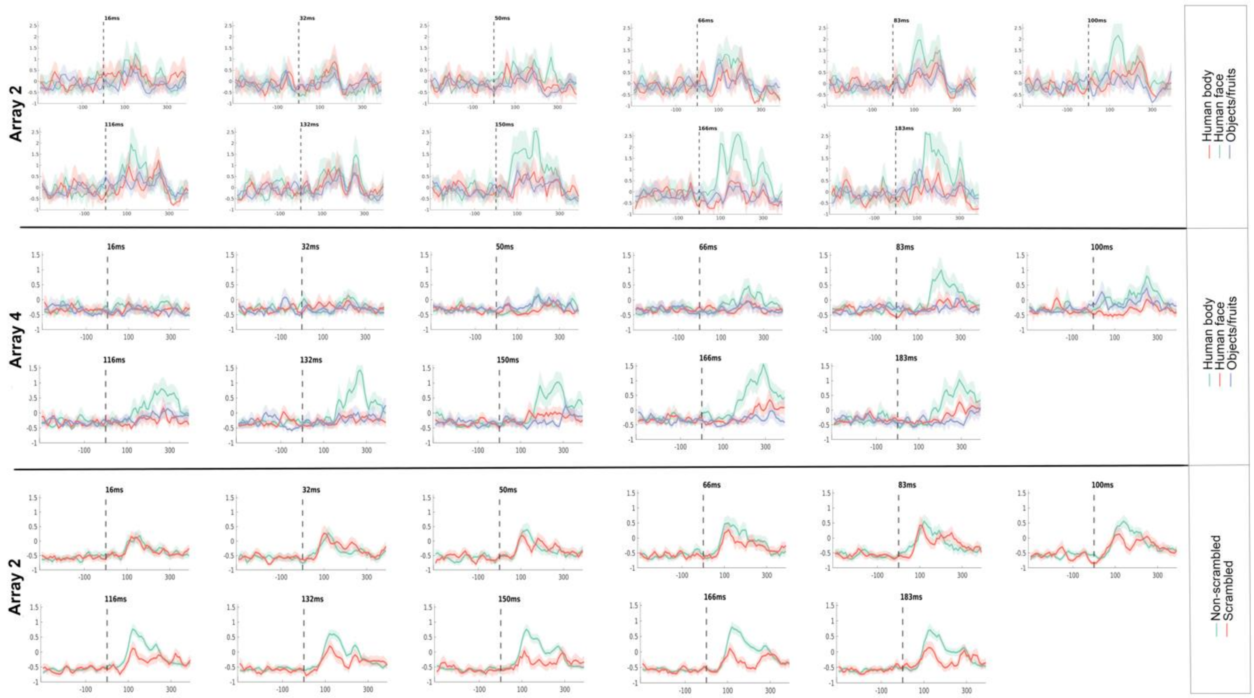

Supplementary Figure S9. Backward masking: average z-normalized response per delay for all visually selective single units (preferred class > all other classes; $p < 0.01$; one-sided permutation test). No distinction is made between perceived and non-perceived target stimuli. The preferred target stimulus is always plotted in green (Array 2: Human faces ; Array 4: Human bodies ; Array 2: Non-scrambled images). In each plot, the x-axis represents time (in milliseconds), while the y-axis represents the average z-normalized net response for all visually selective single units. The dotted line indicates the onset of the target stimulus. Shading represents standard error (N = number of trials x number of visually selective channels).

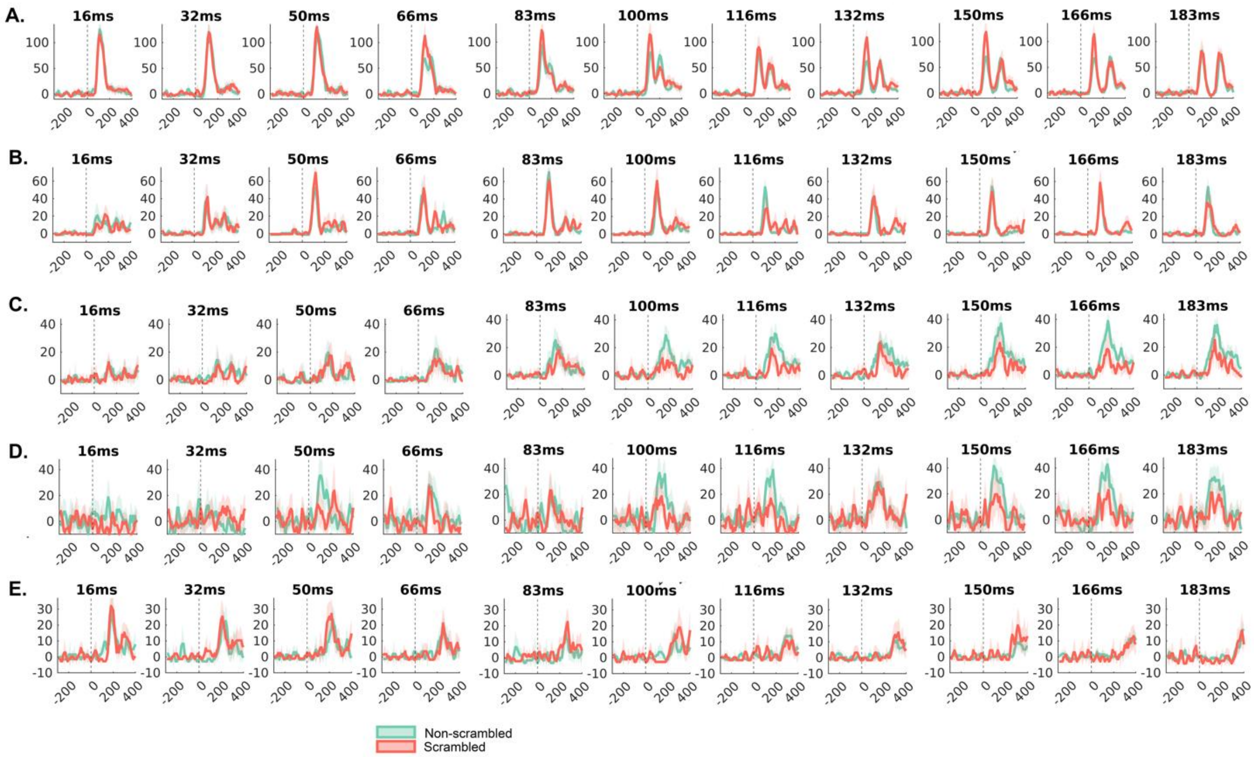

Supplementary Figure S10. Backward masking: net spike rate from example single units. Example neurons from array 2 during backward masking (Non-scrambled vs Scrambled), highlighting the heterogeneity within LO responses. Plots show average net spike rate (± standard error, N = number of trials per condition per delay). **A)** Neuron responds strongly to masking Mondrian and both classes of target stimuli. Both responses integrate with shorter delays. **B)** Neuron responds equally strong to both classes of target stimuli, with only a minimal response to masking Mondrian. Response fades for the shortest delays. **C-D)** Both neurons respond only to the target stimuli, preferring the non-scrambled conditions. Response fades for the shortest delays. **E)** Neuron responds only to masking Mondrian.

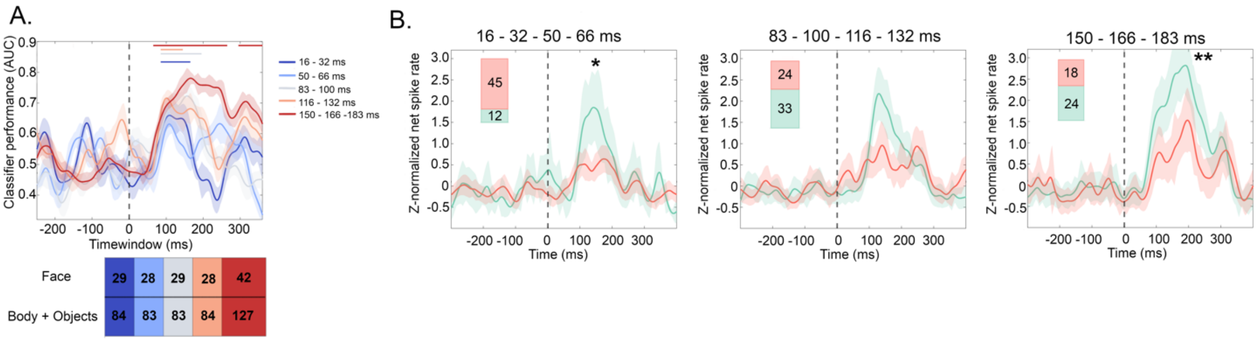

Supplementary Figure S11. Backward masking of categorical stimuli in Array 2. Same plots as displayed in Figures 3A and 3B. **A)** Linear decoder results of the preferred class for different delays, regardless of stimulus perception. Time on x-axis represents middle of 100 ms bins used for decoding. Lines and shading represent the mean ± standard error classification AUC from 10 decoding repetitions. Horizontal bars represent significant decoding performance compared to random label shuffling ($p < 0.001$, permutation test with at least five consecutive significant time points). The bars below the plots show the number of trials per condition. **B)** Average z-normalized spike rate from all channels selective for the preferred class (preferred class > all other classes; $p < 0.01$, one-sided permutation test). Shading signifies standard error. Asterisks indicate significance between perceived and non-perceived target stimuli within the response window ([130-280 ms]) (*$p < 0.05$; **$p < 0.001$; 16-66 ms: $p = 0.018$; 83-132ms: $p = 0.122$; 150-183 ms: $p = 6.28 \times 10^{-4}$; two-sided independent samples test). The bars in the left upper corner of each plot show the number of perceived (green) and nonperceived (red) trials for the preferred class.

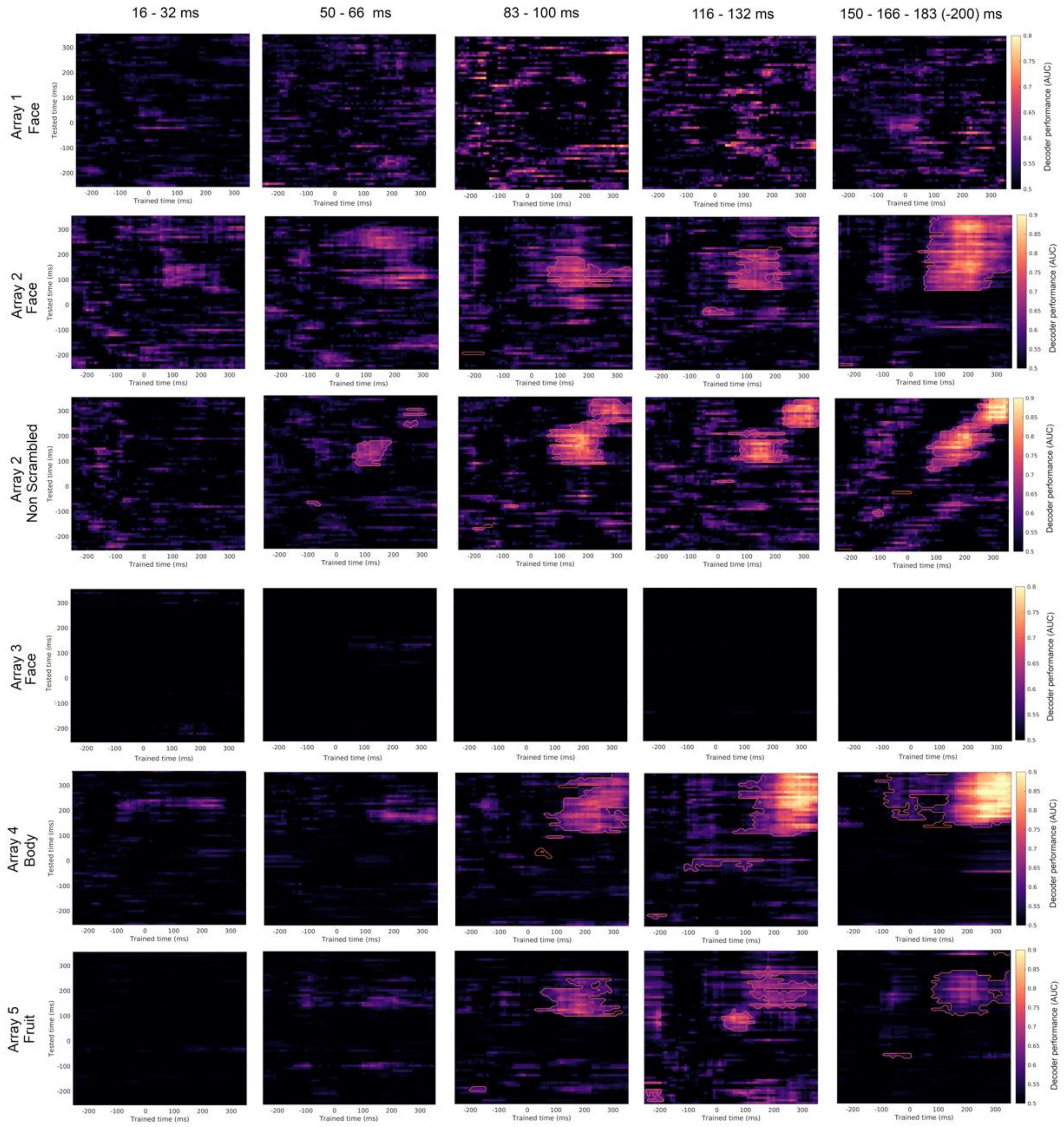

Supplementary Figure S12. Backward masking: decoding performance (AUC) for best decoded category versus all other categories. Plots separated on the y-axis represent different arrays. Best decoded categories were face, face, face, body and fruits/objects for array 1, 2, 3, 4 and 5 respectively. Plots separated on the x-axis represent different delays between target stimulus and masking Mondrian. Within each plot, the x-axis represents the 100 ms bin on which the decoder is trained, while y-axis represents the 100 ms bin on which the decoder is tested. Times on the x- and y-axis represent the middle of the 100 ms interval used for classification. Highlighted contours represent significantly better decoder performance compared to randomly shuffled labels ($p<0.01$; one-sided permutation test). The lack of decoding in array 3 is attributable to low data quality at the day of recording.

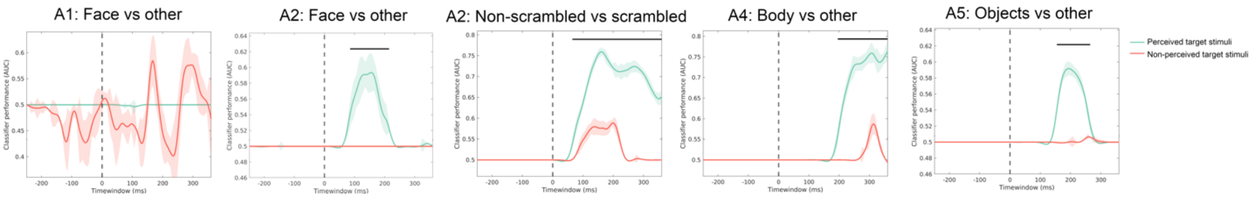

Supplementary Figure S13. Preferred class decoding based on target stimulus perception. Average decoding performance (AUC) is shown for instances where the target stimulus was correctly perceived (green) versus not perceived (red), pooled from all delays. Shading represents the standard error classification AUC from 10 decoding repetitions. Horizontal lines indicate significantly better decoding for perceived compared to non-perceived target stimuli ($p < 0.001$; one-sided permutation test) and are shown only when at least five consecutive bins were significant. In all arrays except A1, classification performance was significantly better when the target stimulus was perceived. However, since all delays were pooled, this perception-dependent classification effect could still be confounded by a larger amount of correctly perceived target stimuli at longer delay intervals. For array 5, objects were the best decodable category, while not eliciting the strongest response (since face and body elicited similarly strong responses).

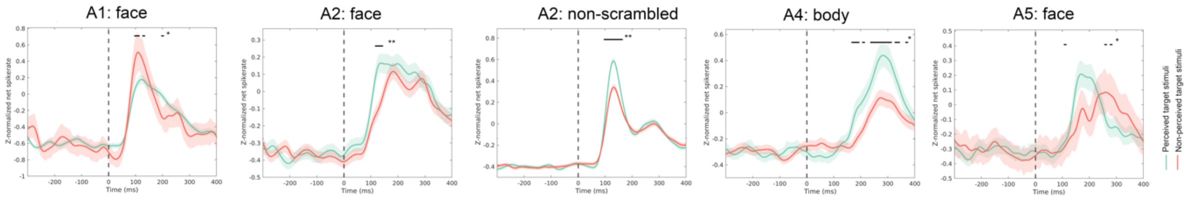

Supplementary figure S14. Backward masking: perceived vs non-perceived target stimuli. Average z-normalized response (± standard error) to the preferred class in case the target stimulus was perceived (red) or not (red). Responses are grouped over all visually responsive channels. Horizontal bars indicate significance between perceived and non-perceived target stimuli (* $p<0.05$, ** $p<0.01$, one-sided permutation test).

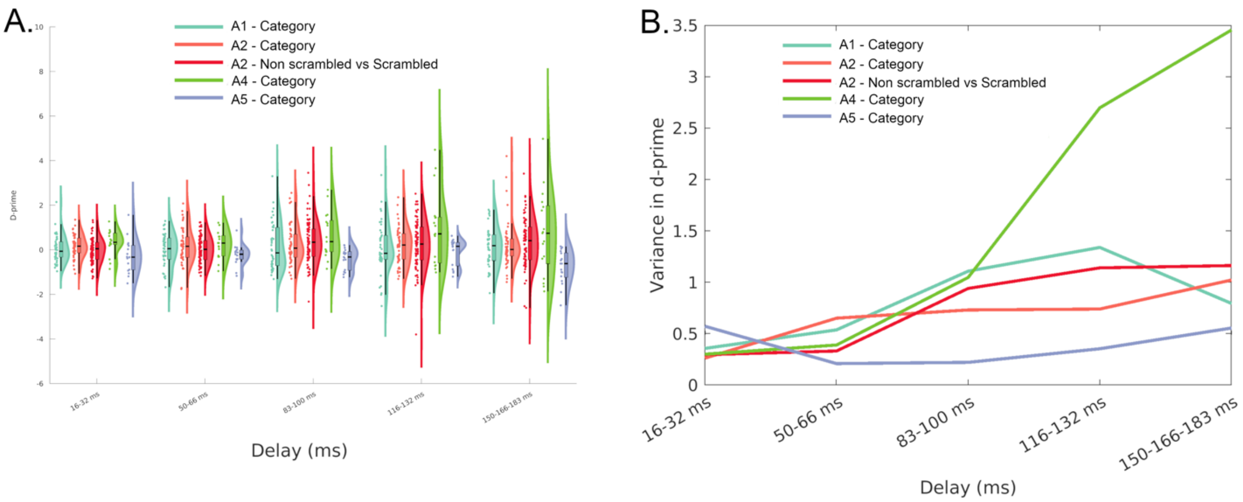

Supplementary Figure S15. Backward-masking: D-prime value. A) Combined scatterplot, boxplot and violin plot from D-prime values for all visually responsive channels per array, separated by delay. D-prime is determined between the best classifiable group and all other stimuli. **B)** Variance of D-prime values for different delays. Note the increasing variance in longer delays for array 4, which is believed to be in a body-patch.

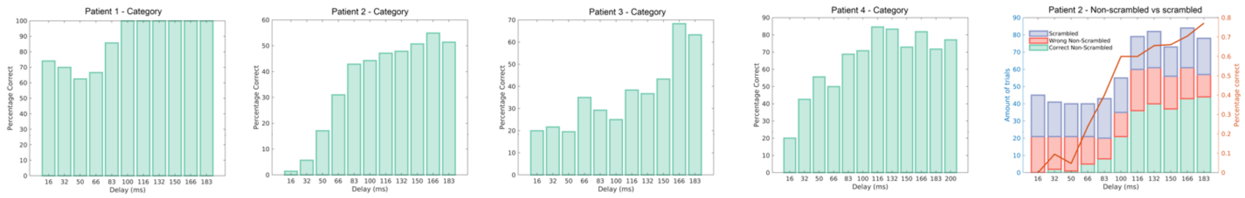

Supplementary Figure S16. Backward masking: accuracy of reported perception as a function of delay. The first four plots display the results from the categorical stimuli that were masked, while the last plot shows the results from masked non-scrambled and scrambled images, which was conducted only on patient 2. In the last plot, the red line indicates the percentage of correct responses, corresponding to the values on the right y-axis.

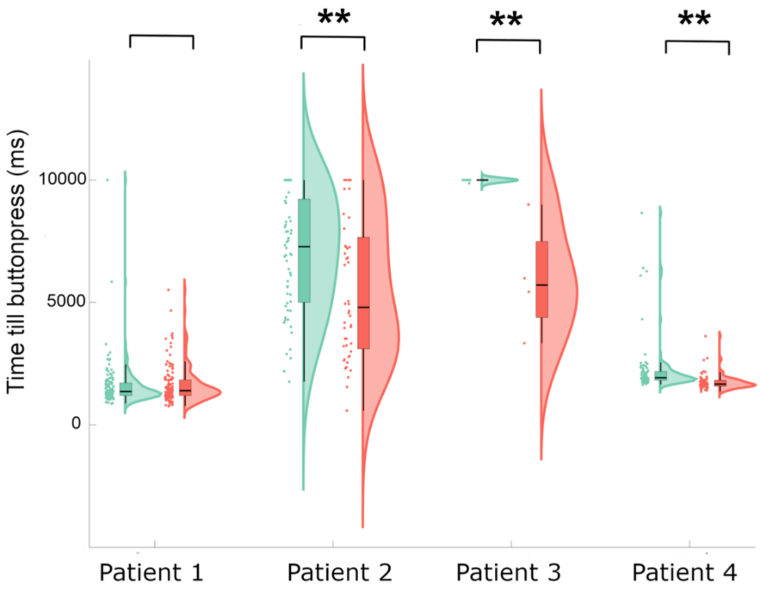

Supplementary Figure S17. Eye Dominance. Combined scatter-, box- and violin plot showing delays between trial onset and the moment of breakage of the continuous flash suppression, the moment that the direction of the arrow is perceived. Box plots indicate median (middle line), 25th, 75th percentile (box) and box limits $\pm$ 1.5 x interquartile range (whiskers). Green indicates arrows presented to left eye, red to the right eye. Asterisks indicate significance level (* $p < 0.05$; ** $p < 0.001$, independent samples t-test).

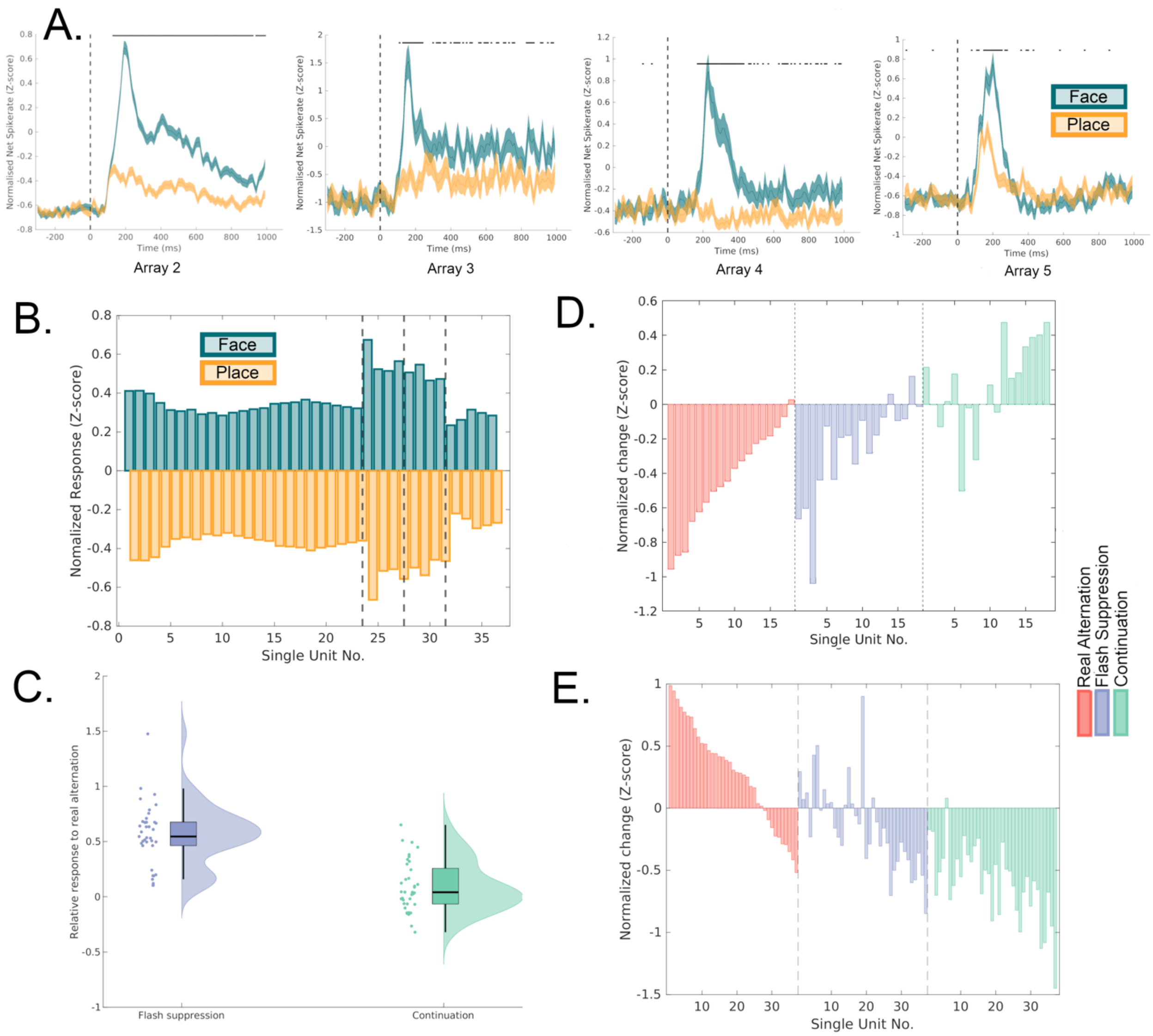

F.

	Face Selective	Follow face percept	Place Selective	Follow place percept
**Array 2**	19	10	2	2
**Array 3**	4	2	3	3
**Array 4**	4	2	0	0
**Array 5**	4	4	0	0

Supplementary Figure S18. Flash Suppression: single-unit selectivity. A) Average z-normalized net spike rate for all face-selective neurons per array in response to the face and place image. Horizontal bars represent significant differences between face and place ($p < 0.01$, one-sided permutation test). Shading

represents standard error. **B)** Z-normalized response to the face and place stimulus from all 37 face-selective single units. Vertical dotted lines separate different arrays (A2: SUA 1–24; A3: SUA 25–28; A4: SUA 29-32; A5: SUA 33-37). **C)** Combined scatter, box and violin plot of relative responses for all face and place selective SUA for flash suppression (non-preferred to preferred stimulus) and continuation of non-preferred stimulus, compared to real alternation (1 = response to real alternation, 0 = baseline activity during fixation period). **D)** Change in net spike rate (z-normalized) for all 18 face-selective single units classified as either sustained or inhibitory responders after the start of phase 2, when the face stimulus was presented in phase 1. During Continuation, the face stimulus persists with no change in perception. In Real Alternation and Flash Suppression conditions, perception shifts from face to place. Each bar represents a different single unit. All single units within the 3 conditions are ranked in ascending order based on values obtained from the Real Alternation condition. **E)** Change in net spike rate (z-normalized) for all face selective single units. During Continuation, the place stimulus persists with no change in perception. In Real Alternation and Flash Suppression conditions, perception shifts from place to face. Each bar represents a different single unit. All single units within the 3 conditions are ranked in descending order based on values obtained from the Real Alternation condition. **F)** Table with number of face and place selective SUA per array that modulate activity according to the percept.

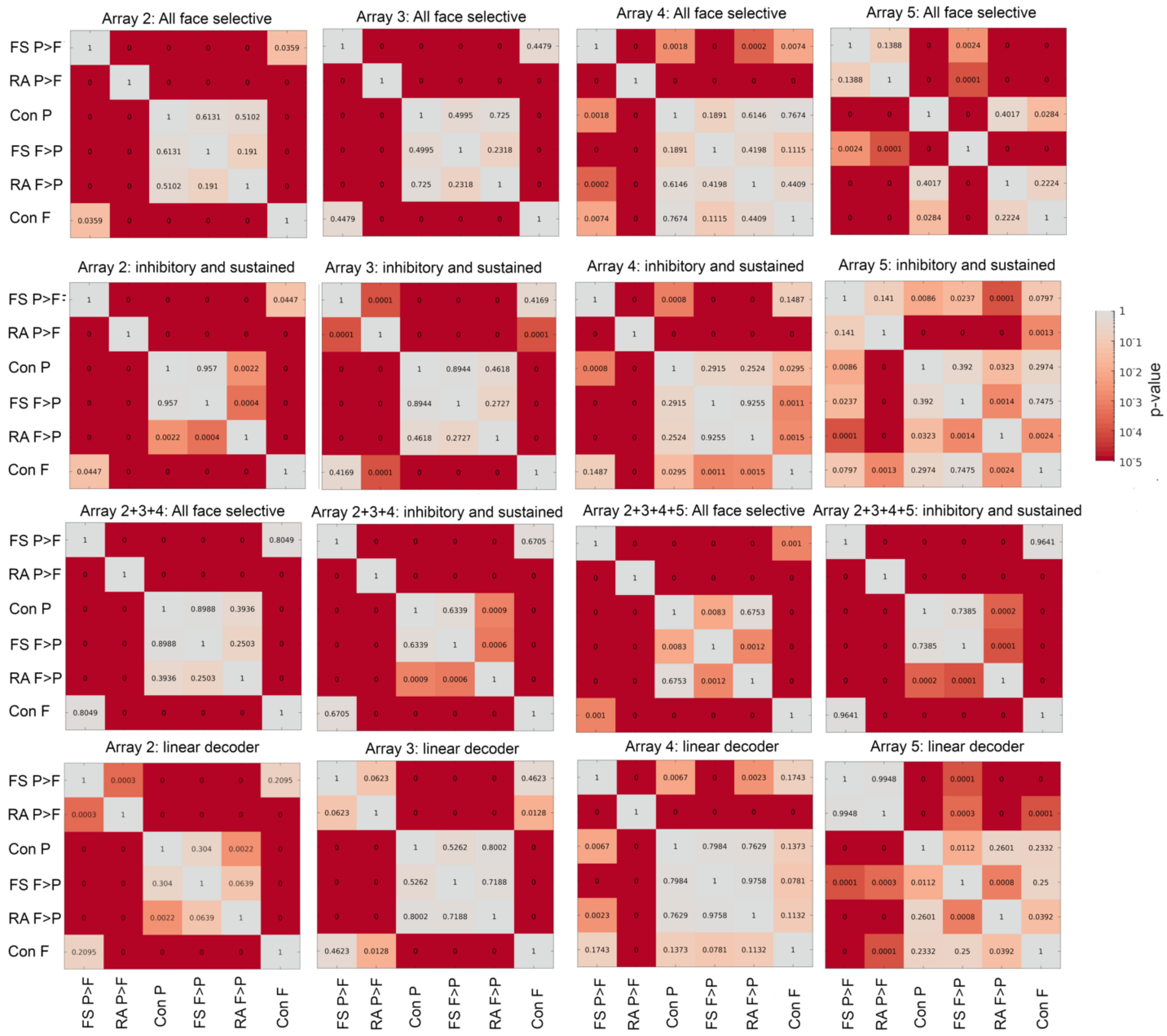

Supplementary Figure S19. Flash suppression: significance levels between different paradigms during phase 2. P-values (two-sided permutation test) during the second phase of the flash suppression experiment within the response window ([130 280], [130 280], [200 350] and [140 290] for array 2 to 5 respectively). The first 3 rows compare the z-normalized net responses. Bottom row compares decoder results. Top row shows all face-selective SUA, second row shows all inhibitory and sustained responders from face-selective SUA, third row shows the combination of arrays for all face-selective SUA as well as inhibitory and sustained responders. Values of “0” indicate $p < 0.0001$.

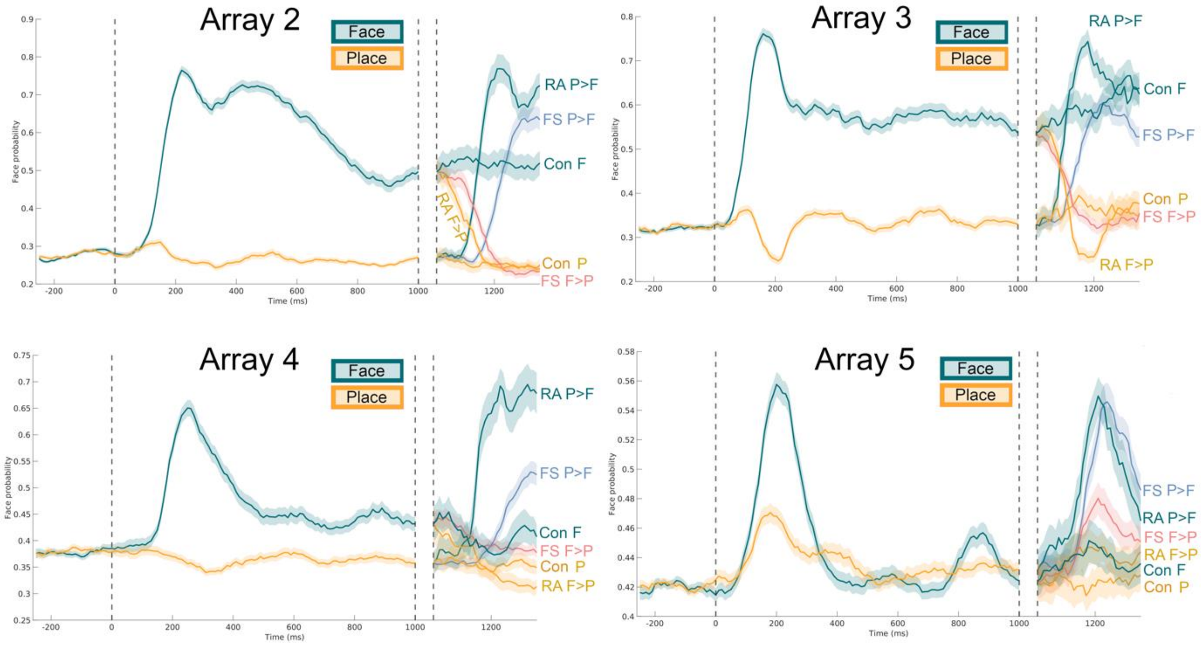

Supplementary Figure S20. Flash suppression: linear decoder. Average decoder face probability for all conditions during phase 1, green and yellow plots are the average prediction for face and place, respectively. During phase 2, average prediction is made for each paradigm separately. Green and yellow indicates face and place perception respectively, obtained through real alternation (RA) or continuation of the initial stimulus (Con). Blue and red indicate flash suppression (Blue: FS P→F; Red: FS F→P). Each line plot during phase 2 is annotated with the paradigm it represents. Double dotted vertical lines separate phase 1 from phase 2. Shading represents standard error ($N_{decoders}$ = number of trials per paradigm).

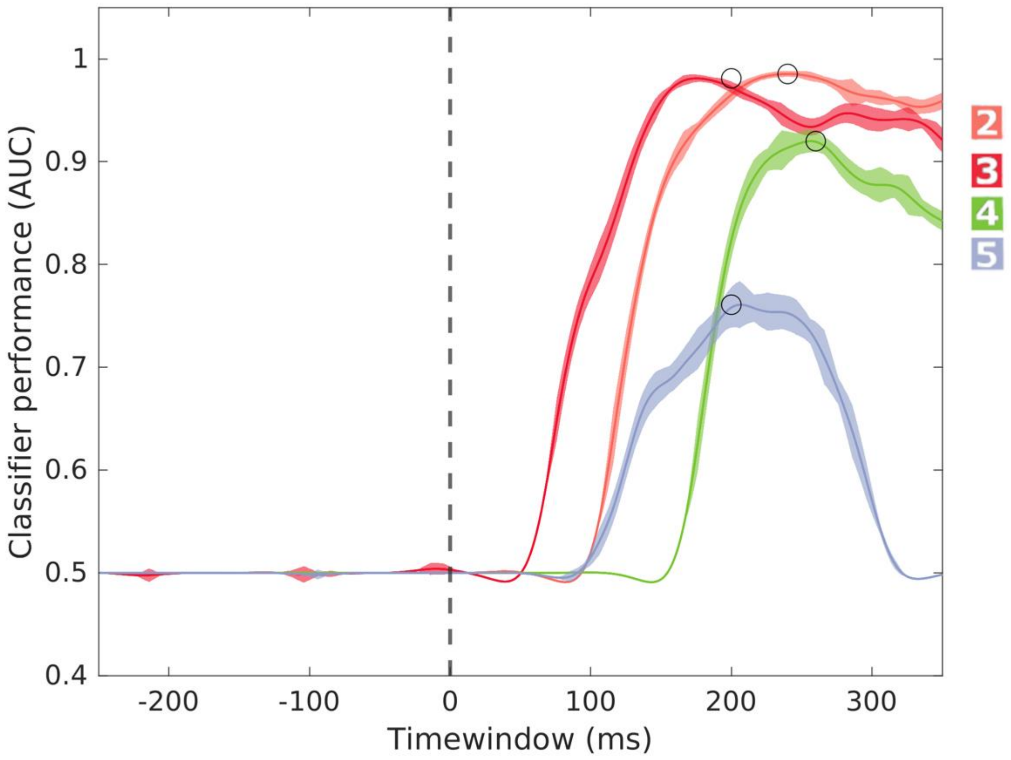

Supplementary Figure S21. Flash Suppression: classifier performance face versus place. Average decoding performance over time (± standard error) between face and place stimulus, using SUA. A logistic regression was trained on 80% of the data by 10-fold cross-validation (see Methods) and tested on the remaining 20% of trials. Times on the x-axis represent the middle of the 100 ms interval used for classification. The interval with highest AUC (the 'best bin') is annotated with a circle, and was used as the training interval to classify individual trials from the flash suppression experiment by Leave-One-Out (LOO) cross-validation. Colored numbers indicate array number.

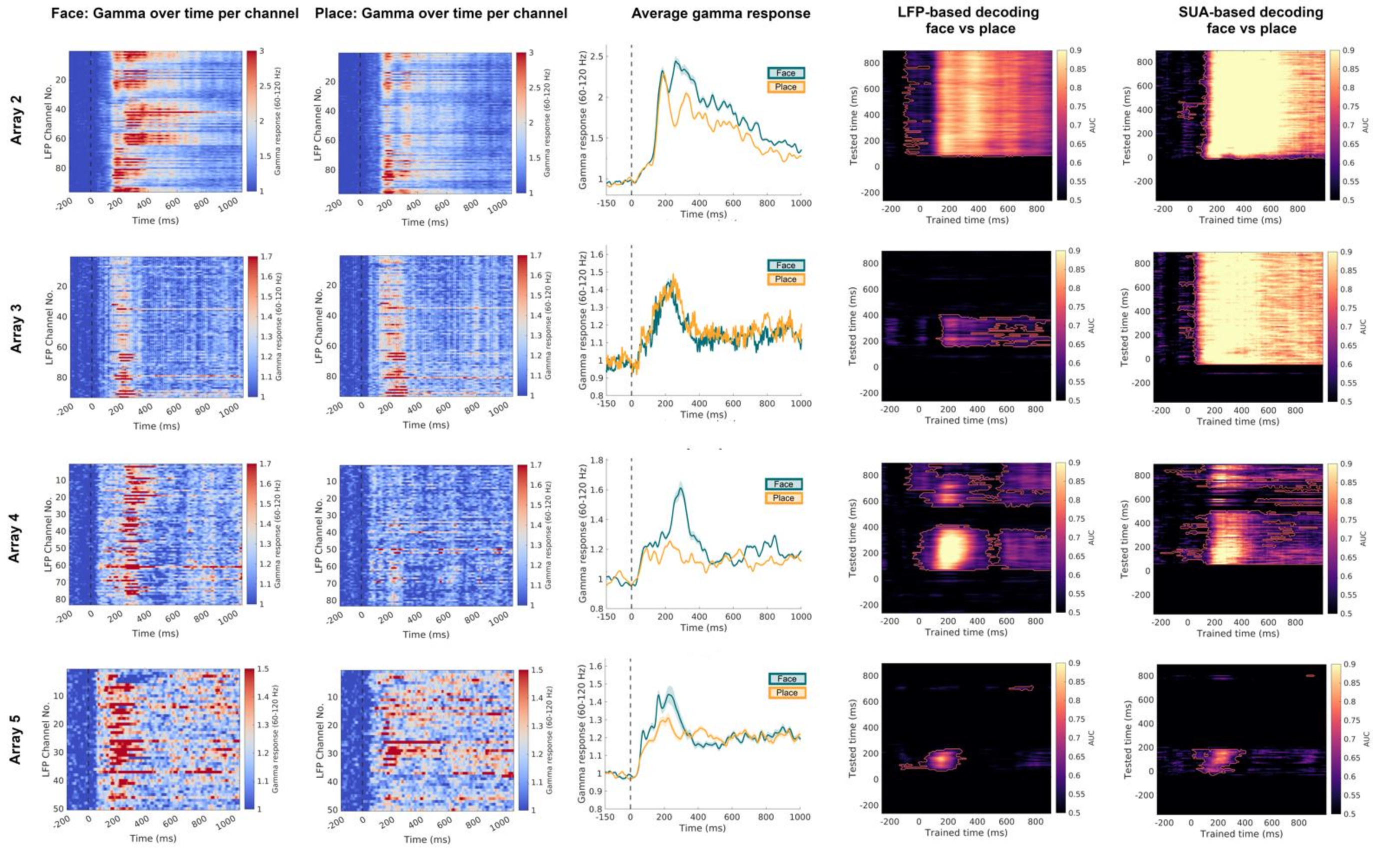

Supplementary Figure S22. Flash Suppression: LFP response (gamma filtered between 60 and 120 Hz) during phase 1 (monocular stimulus presentation). Rows differentiate between different arrays. First 2 columns visualize the normalized gamma response (compared to baseline) per visually responsive channel (y-axis) for the face (first column) and place stimulus (second column). Nearly all visually responsive channels maintain an elevated gamma activity compared to baseline for the whole 1000 ms of face stimulus presentation (array 2: 96/96 ; array 3: 92/93 ; array 4: 81/81 ; array 5: 50/50). Third column visualizes the average gamma response ($\pm$ standard error) over all visually responsive channels. Fourth and fifth row visualize cross-temporal separability/decodability between the face and place stimulus using gamma activity (fourth column) and single-unit activity (fifth column). The orange contour represents significant decoding compared to random label shuffling ($p<0.01$, independent samples t-test).

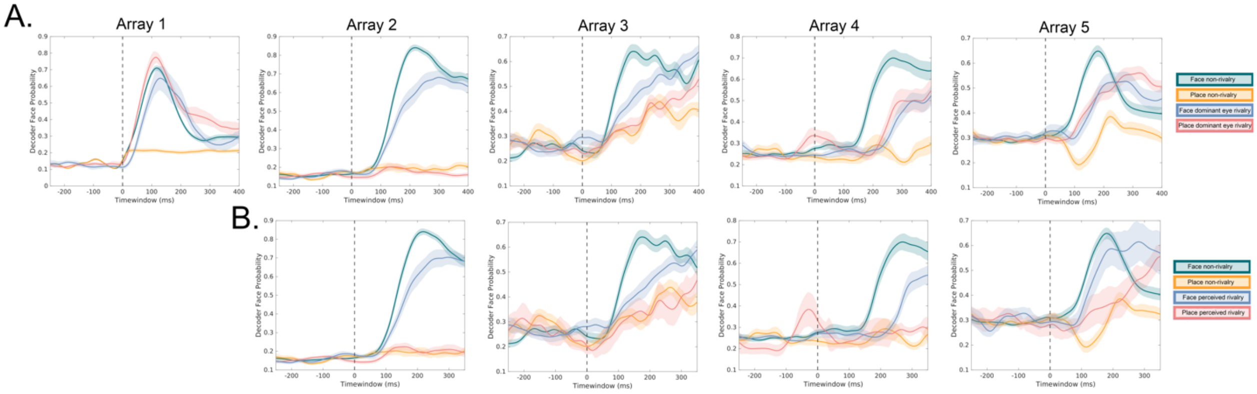

Supplementary Figure S23. Binocular rivalry: decoding after stimulus onset. In both A and B, the graphs show the average probability over time that a face was presented for separate conditions after stimulus onset. Baseline decoder activity, before stimulus onset (Time = 0 ms), represents the face probability during the 300 ms fixation period before each trial. Shading represents standard error (N = number of trials per condition). **A)** The green and red graphs represent conditions where the face and place images were non-rivalrous presented binocularly. The blue and purple graphs depict rivalrous conditions, not taking the perceived image into account. In array A1, the decoder identified the presence of a face during rivalrous conditions, regardless of whether the face was presented to the left or right eye. In A2, the classifier gave high face probability only when the face was presented to the dominant eye. For arrays 3, 4, and 5, the decoder produced intermediate results for both rivalrous conditions (face in dominant or non-dominant eye) compared to non-rivalrous face and place-perception. In array 3, but not in arrays 4 and 5, the decoder produced higher probabilities for face perception when the face was presented to the dominant eye compared to the non-dominant eye, regardless of reported perception ($p < 0.05$, two-sided permutation test). **B)** The green and red graphs represent conditions where the face and place images were non-rivalrous presented binocularly. The blue and purple graphs depict conditions under rivalry, where the face (blue) or place (purple) was perceived as first image. Array 1 is not presented because the patient did not correctly categorize images during rivalry.

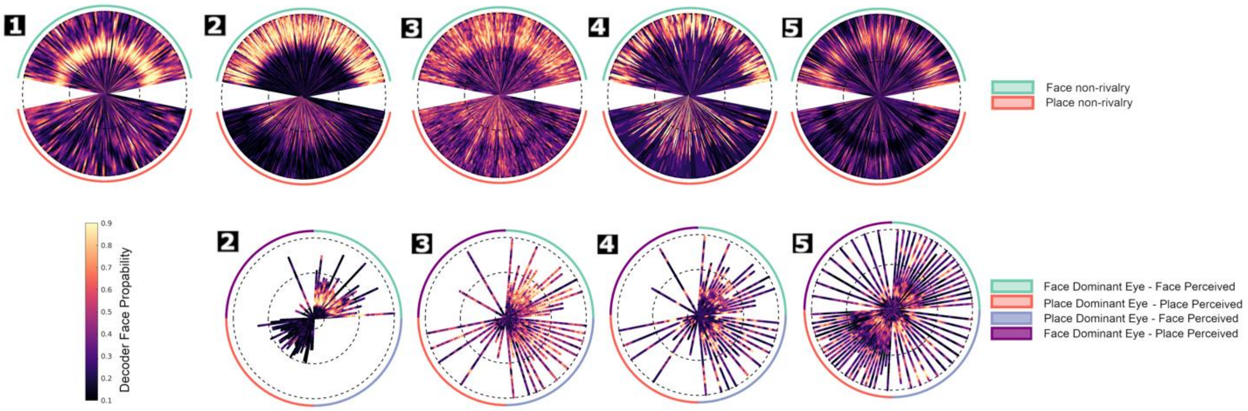

Supplementary figure S24. Trial by trial face probability prediction after stimulus onset. Top) Decoding for non-rivalrous face and place perception. Inner dotted circle marks stimulus onset, and the outer dotted circle marks 400 ms post-onset. Each line, originating from the center, represents a perceptual shift towards face (green) or place (red). Numbers indicate array number. **Bottom)** Decoding for rivalrous face and place perception, considering the eye (dominant or non-dominant) in which each stimulus was presented and the actual perceptual report. Inner dotted circle indicates stimulus onset, whereas outer circles mark 1000 ms and 2000 ms post-onset. The length of each line corresponds to the timing of the button. If the button press exceeds 2000 ms after stimulus onset, the line is capped at 2000 ms (outer circle). All trials are linearly spaced and plotted in chronological order as appeared during the experiment.

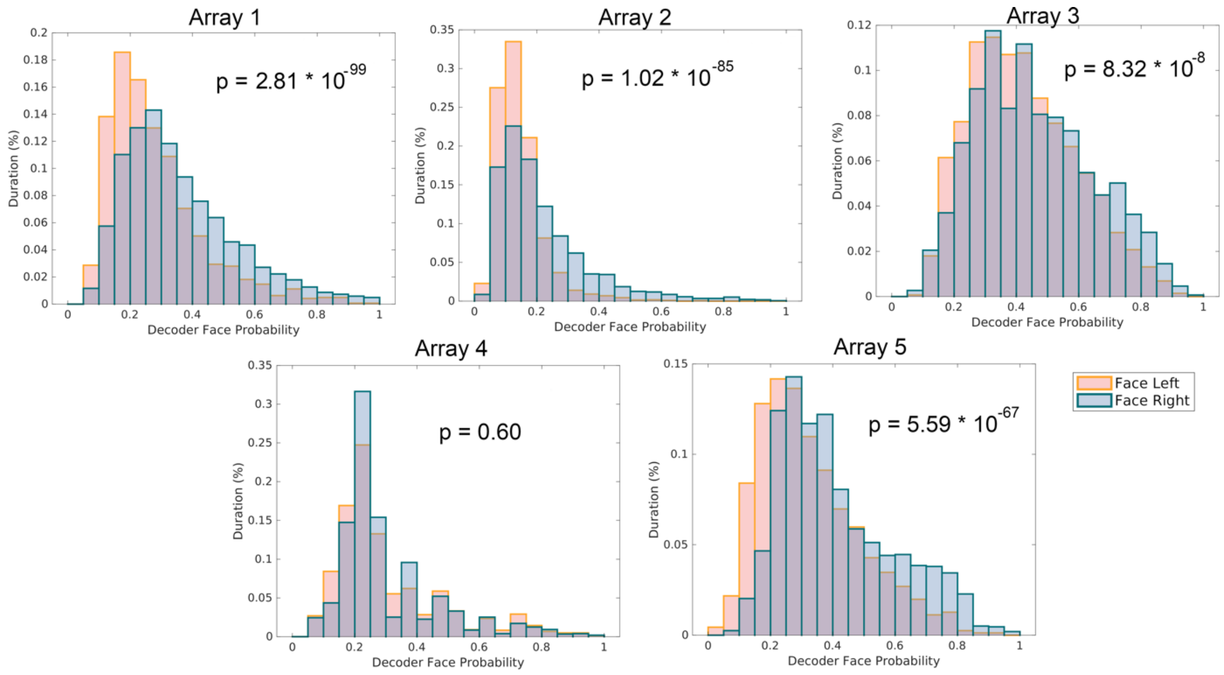

Supplementary Figure S25. Binocular rivalry. Histograms displaying the relative frequency of bins across all rivalrous trials, each corresponding to a specific probability of face/place perception (face = 1, place = 0). The histogram bin size is set to 0.05 (range: [0 : 0.05 : 1]). Blue bars represent trials where the face stimulus was shown to the right eye, while red bars represent trials where the place stimulus was presented to the right eye. In arrays 2, 3, 4, and 5, the right eye also served as the dominant eye. P-value indicates significance between green and red bars (independent samples t-test).

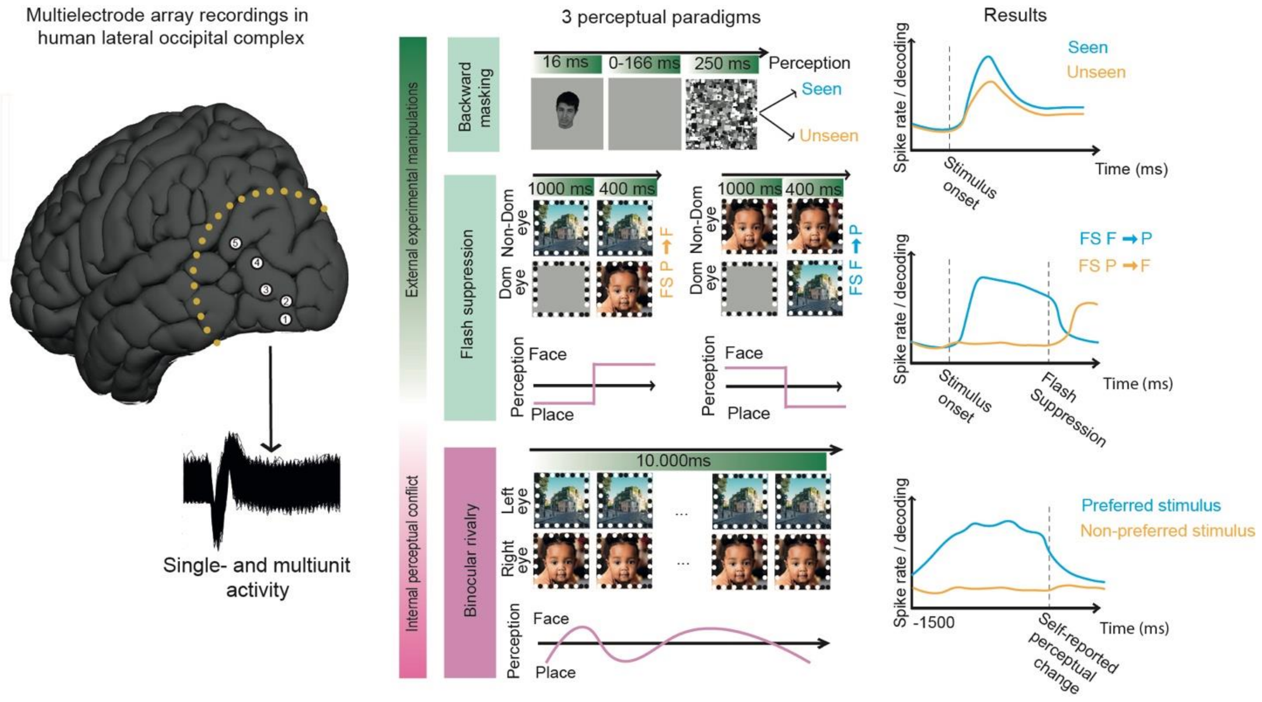

Supplementary Figure S26. Summary of the main findings. We recorded single- and multiunit activity from five 96-channel multielectrode arrays implanted in the lateral occipital complex of four human patients. Three perceptual paradigms were used in which subjective perception varied despite identical visual stimulation. These perceptual dissociations arose from either external experimental manipulations (backward masking and flash suppression) or internal perceptual conflict (binocular rivalry). Our findings show that perceived stimuli elicited stronger neural responses during the feedforward propagation of information (backward masking) and that neural activity predominantly reflected the perceived, rather than the perceptually suppressed, stimulus (flash suppression and binocular rivalry). Neural responses were weaker during dichoptic presentation compared to rivalrous viewing (flash suppression). Moreover, neural activity began to predict the upcoming percept approximately 1.5 seconds before subjects reported a perceptual switch (binocular rivalry). The cortical brain rendering is adapted from Bougou, V., Vanhoyland, M., Bertrand, A. et al. Neuronal tuning and population representations of shape and category in human visual cortex. Nat Commun 15, 4608 (2024). Stimuli used during backward masking were first published in Popivanov, I. D., Jastorff, J., Vanduffel, W. & Vogels, R. Stimulus representations in body-selective regions of the macaque cortex assessed with event-related fMRI. Neuroimage 63, 723–741 (2012), Copyright Elsevier.

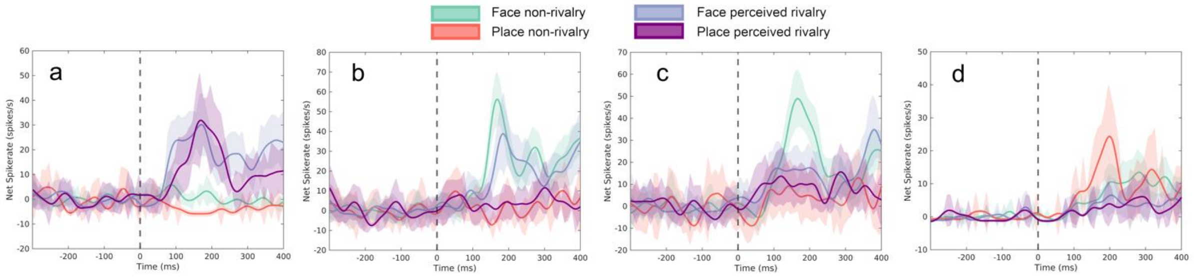

Supplementary figure S27. Binocular rivalry: example neurons from array 2. Average spike rate after stimulus onset from example neurons, highlighting that not all neurons modulate activity according to perception. Horizontal dotted line represents stimulus onset. Shading represents standard error. **a)** Only response to rivalry trials, thereby responding to the visual input rather than perceived image. **b)** Face selective neuron that modulates activity according to perception. **c and d)** Face **(c)** and place **(d)** selective neurons that don’t modulate activity according to perception.

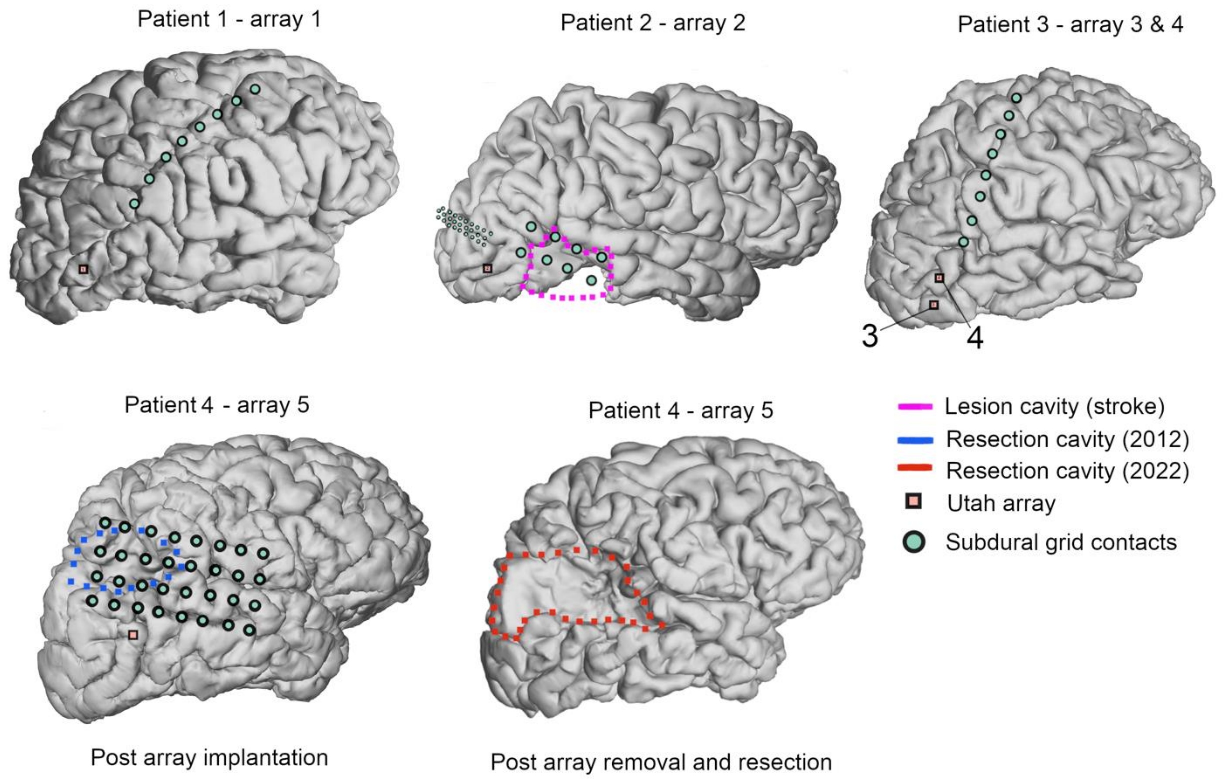

Supplementary figure S28. Array locations in all patients. Anatomical depiction of array position, subdural grid contacts and previous cavity (stroke cavity for patient 2, resection cavity for patient 4). For patient 4**,** post-operative cortical rendering after electrode removal and more extensive resection is included. Red dotted line highlights the performed resection from the presumed epileptogenic zone (PEZ) in patient 4.

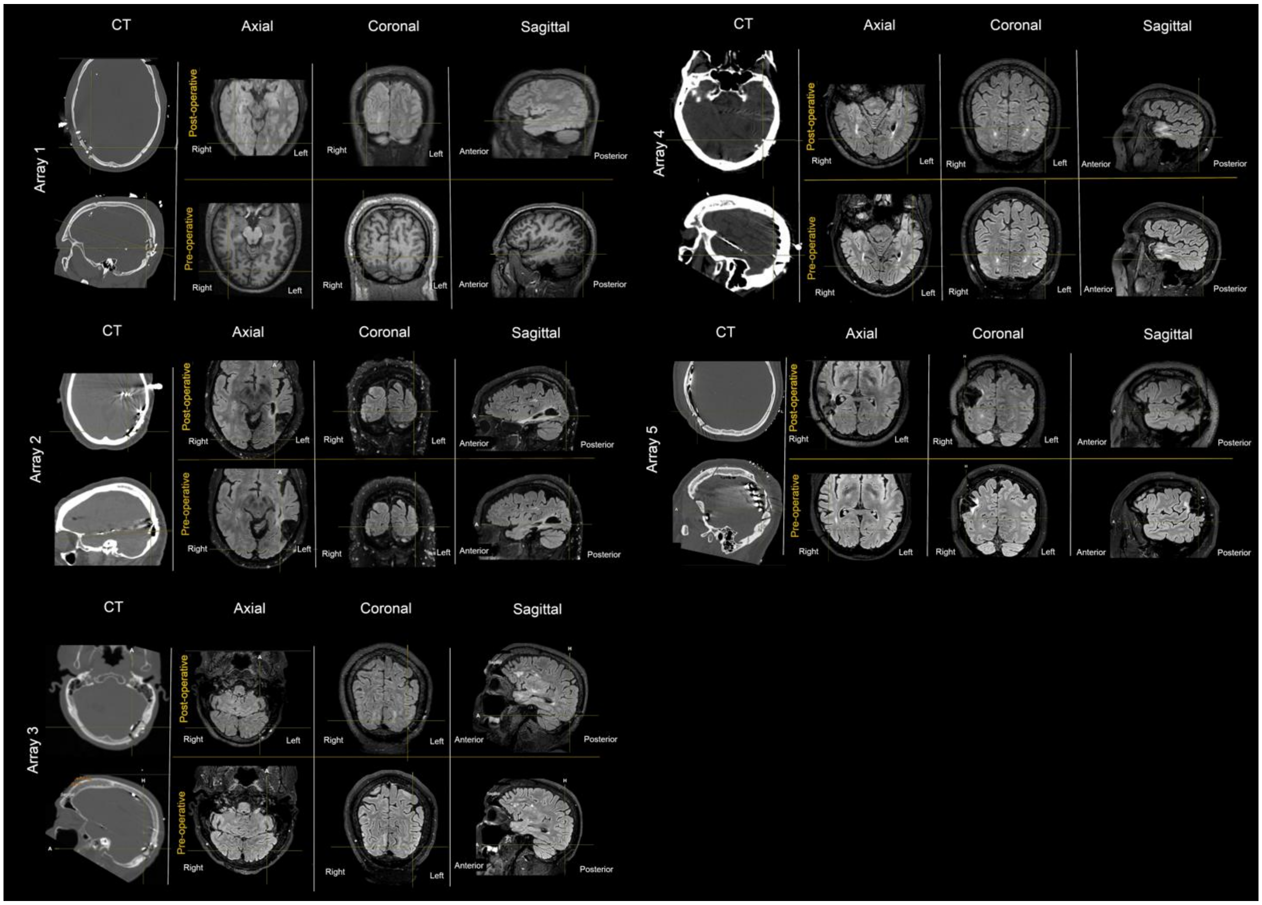

Supplementary figure S29. Array localization in all 4 patients. Axial, coronal, and sagittal slices of pre-implantation and post-explantation MRI (FLAIR) scans are presented. Pre-implantation MRI, post-explantation MRI, and post-implantation/pre-explantation CT scans were co-registered using Brainlab Elements software. The exact location of the array was determined based on CT imaging. Notably, no lesions were visible on the post-explantation MRI in the area where the array was inserted. Note that for array 5, an additional resection occurred during electrode removal, resulting in an enlargement of the previously existing resection cavity. There was no pre-operative FLAIR available with enough slices for patient 1, so a T1 was used instead.

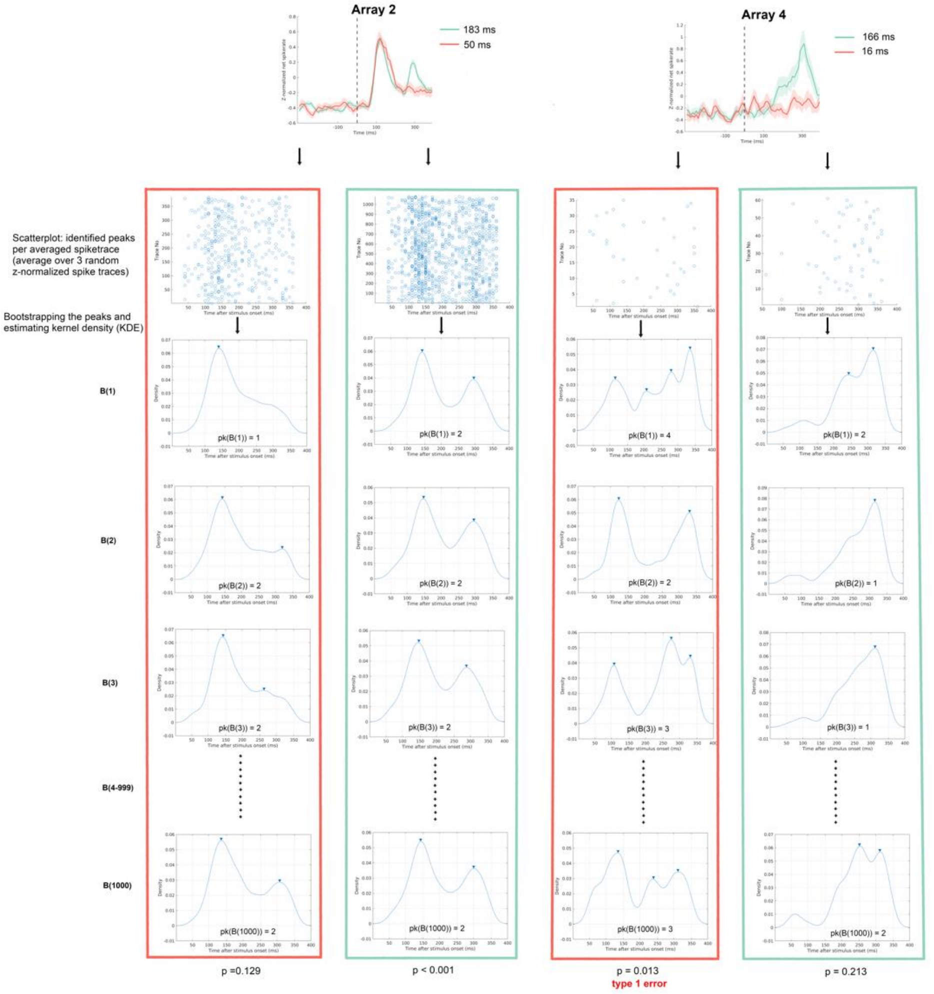

Supplementary figure S30. Backward masking: Silverman's test and the risk of Type I errors. The upper plots show the average z-normalized spike rate for specific delays in array 2 (variation 2) and array 1 (variation 1). To reduce noise, individual traces contributing to the total average were shuffled and grouped in sets of three, with the average taken for each group. Peaks were identified in all averaged traces (scatter plot), after which the timestamps corresponding to the identified peaks were bootstrapped 1000 times with replacement. For each bootstrap sample, the kernel density estimate (KDE) was computed, and the number of peaks in the KDE was determined. Note the significant p-value observed in the 16 ms delay group in array 4, despite the absence of a clear visual response, indicating a Type I error.

Supplementary figures from the point by point answers to the reviewers:

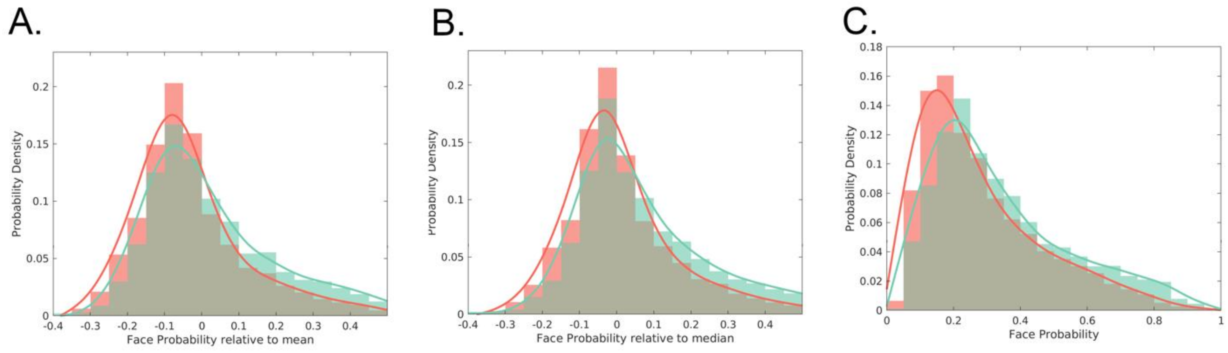

Supplementary figure S31. Probability density function of decoder results for arrays 2, 3, 4, and 5 during binocular rivalry. Face perception probability is higher when the face stimulus is presented to the dominant eye (green) compared to the nondominant eye (red). In A, the mean decoder probability was subtracted for each array individually (p = 7.78 x 10^{-92} ; two-sided independent samples t-test). In B, the median decoder probability was subtracted for each array individually (p = 4.97 x 10^{-94} ; two-sided independent samples t-test). In C, no subtraction was done per array (p = 5.87 x 10^{-75} ; two-sided independent samples t-test).

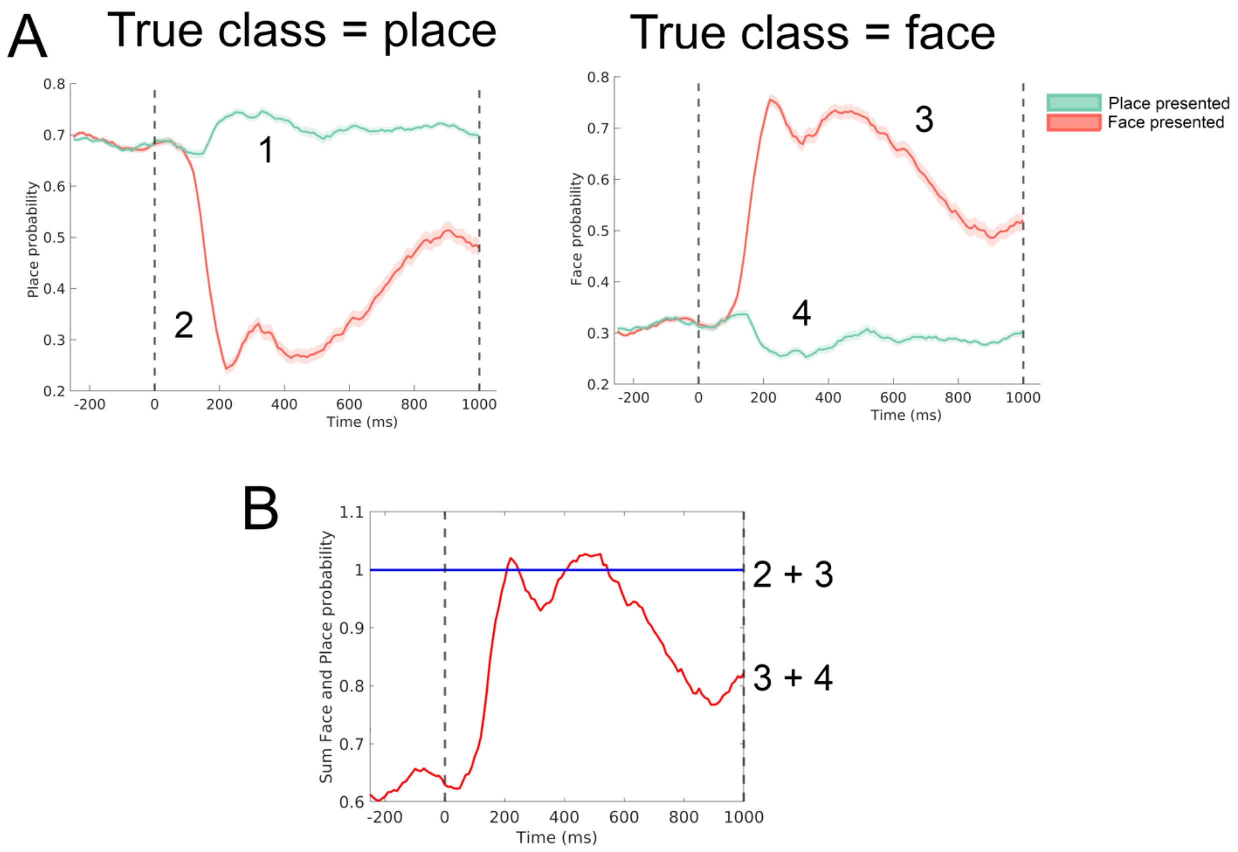

Supplementary figure S32. Effect of changing the true class for decoding. A) Place probability (decoder trained with true class = place; left) and face probability (decoder trained with true class = face; right) when a place (green) and face (red) is shown during phase 1 of flash suppression (array 2). Lines and shading represent the mean ± standard error classification AUC from 10 decoding repetitions. **B)** The total probability that a stimulus belongs to either class always equals 1 (blue line) (e.g. probability that a face stimulus is a place (2) + probability that a face stimulus is a face (3)). The total face probability over time (red line) does not constantly equal 1 (e.g. probability that a face is a face (3) + probability that a place is a face (4)), but will approximate 1 at the bin on which the decoder was trained (best bin).

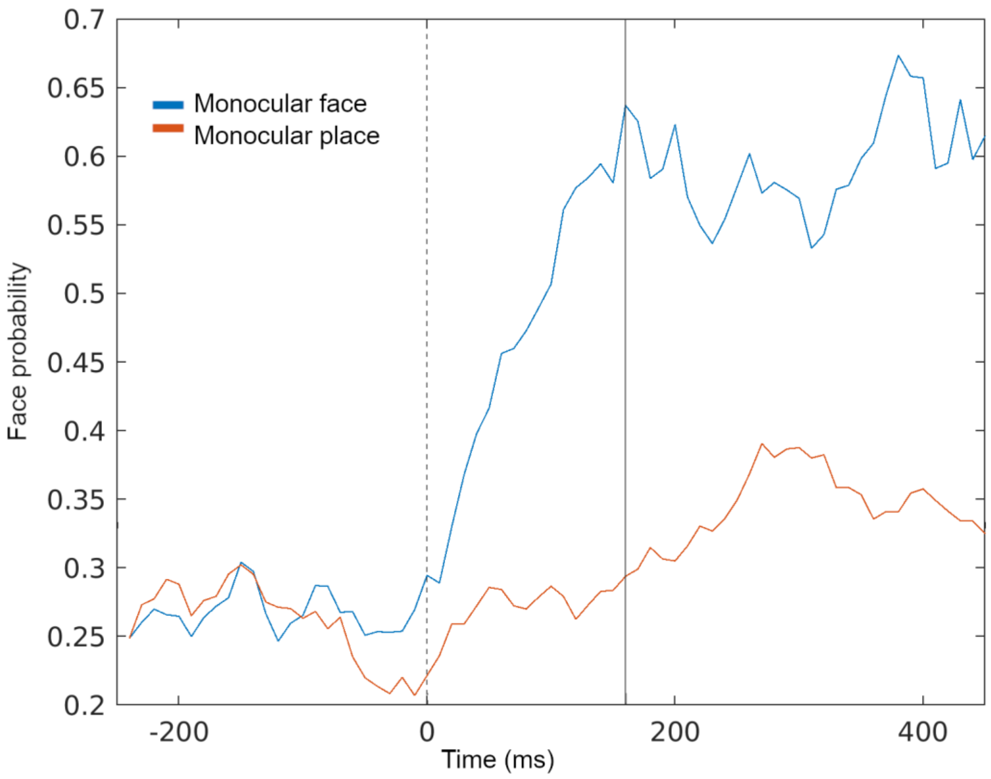

Supplementary figure S33. Face probability after stimulus onset during monocular face and place presentation during the binocular rivalry experiment (array 3). The decoder was trained on the most discriminative bin to separate face from place responses (full vertical line, 160 ms). Face probability also increases slightly from baseline during monocular place presentation. Vertical dotted line represents stimulus onset. Time on the x-axis represent the middle of the 100 ms bin used for decoding.
