# Supplementary material for: Single-neuron correlates of visual consciousness in human lateral occipital complex
Source: Nat Commun. 2025 Dec 15;16:11008. doi: 10.1038/s41467-025-67077-w (PMC12705761; doi:10.1038/s41467-025-67077-w)
Supplement: Supplementary file 2 — Reporting Summary [file 41467_2025_67077_MOESM2_ESM.pdf]

nature portfolio

Corresponding author(s): Michaël Vanhoyland

Last updated by author(s): Sep 28, 2025

Reporting Summary

Nature Portfolio wishes to improve the reproducibility of the work that we publish. This form provides structure for consistency and transparency in reporting. For further information on Nature Portfolio policies, see our Editorial Policies and the Editorial Policy Checklist.

Statistics

For all statistical analyses, confirm that the following items are present in the figure legend, table legend, main text, or Methods section.

n/a	Confirmed	
☐	☒	The exact sample size (*n*) for each experimental group/condition, given as a discrete number and unit of measurement
☐	☒	A statement on whether measurements were taken from distinct samples or whether the same sample was measured repeatedly
☐	☒	The statistical test(s) used AND whether they are one- or two-sided<br>*Only common tests should be described solely by name; describe more complex techniques in the Methods section.*
☒	☐	A description of all covariates tested
☐	☒	A description of any assumptions or corrections, such as tests of normality and adjustment for multiple comparisons
☐	☒	A full description of the statistical parameters including central tendency (e.g. means) or other basic estimates (e.g. regression coefficient) AND variation (e.g. standard deviation) or associated estimates of uncertainty (e.g. confidence intervals)
☐	☒	For null hypothesis testing, the test statistic (e.g. *F*, *t*, *r*) with confidence intervals, effect sizes, degrees of freedom and *P* value noted<br>*Give P values as exact values whenever suitable.*
☒	☐	For Bayesian analysis, information on the choice of priors and Markov chain Monte Carlo settings
☒	☐	For hierarchical and complex designs, identification of the appropriate level for tests and full reporting of outcomes
☒	☐	Estimates of effect sizes (e.g. Cohen's *d*, Pearson's *r*), indicating how they were calculated

Our web collection on statistics for biologists contains articles on many of the points above.

Software and code

Policy information about availability of computer code

Data collection

We recorded neural activity using a 96-channel array (Utah ArrayTM – Blackrock Neurotech, USA) from day 1, the day after implantation, until the day before electrode removal. The signal was digitally amplified using a Cereplex M head stage from Blackrock Neurotech, connected to a 128-channel neural signal processor (NeuroPort), and sampled at 30kHz using Central Software. We applied a 750 Hz high-pass filter to isolate spiking activity, with the multi-unit detection threshold set at -3 standard deviations of the signal. Signal quality varied during the immediate postoperative period, leading to fluctuations in the number of responsive and selective channels across recording sessions. Spike sorting was performed offline (Offline Sorter 4, Plexon, TX).

Data analysis

We analyzed data using custom-written MATLAB R2023b (MathWorks, Natick, MA, USA) scripts. FreeSurfer was used to generate cortical 3D renderings of the subjects' pial surfaces. The precise electrode location on the cortical surface and the MNI coordinates were determined via iElectrodes Software. LFP's were filtered using a combined spectral and spatial filter (Zapline), notch-filtered (50, 100 and 150 Hz) and bandpass filtered between 1 and 450 Hz using a linear-phase finite impulse response (FIR) filter applied in both forward and reverse directions. For every trial, the time-frequency power spectrum was calculated using Morlet's wavelet analysis techniques, with a spectrotemporal resolution of 7. The net spike rate was obtained by subtracting baseline spikerate from the whole trial recording (300 ms before stimulus onset). Mean and standard deviations to z-normalize spike rates were calculated on the response window (selectivity latency till selectivity latency + 150 ms), which differed between arrays. We trained logistic regression classifiers with L1 regularization (LASSO) and C=0.1 in MATLAB. For the 'Non-scrambled vs Scrambled', 'Category' and 'Backward Masking' experiment, the classifier was trained on 80% of the data using 10-fold cross validation, after which the AUC was estimated by testing on the remaining 20% of trials. For 'Flash Suppression' and Binocular Rivalry', we used LOO-cross validation.

For manuscripts utilizing custom algorithms or software that are central to the research but not yet described in published literature, software must be made available to editors and reviewers. We strongly encourage code deposition in a community repository (e.g. GitHub). See the Nature Portfolio guidelines for submitting code & software for further information.

Data

Policy information about availability of data

All manuscripts must include a data availability statement. This statement should provide the following information, where applicable:

- Accession codes, unique identifiers, or web links for publicly available datasets
- A description of any restrictions on data availability
- For clinical datasets or third party data, please ensure that the statement adheres to our policy

Data availability
The raw data have been deposited in the figshare repository at https://doi.org/10.6084/m9.figshare.28319411. The processed data are available at Code Ocean. The underlying data for all figures are provided as a Source Data file.

Code availability
All analyses for the individual experiments (LO localizer stimuli: scrambled vs. non-scrambled, Category Selectivity, Backward Masking, Flash Suppression, and Binocular Rivalry) are available on Code Ocean, organized as separate capsules for each experiment at https://doi.org/10.24433/CO.0628053.v1, https://doi.org/10.24433/CO.2303025.v2, https://doi.org/10.24433/CO.7059539.v1, https://doi.org/10.24433/CO.4388145.v1 and https://doi.org/10.24433/CO.5851818.v1. Each analysis can be fully reproduced within the Code Ocean environment with a single click, automatically generating the results, datasets and figures presented in this work.

Research involving human participants, their data, or biological material

Policy information about studies with human participants or human data. See also policy information about sex, gender (identity/presentation), and sexual orientation and race, ethnicity and racism.

Reporting on sex and gender	Four patients with refractory epilepsy: male 24 years, female 55 years, female 58 years, male 29 years.
Reporting on race, ethnicity, or other socially relevant groupings	Race was not mentioned in the manuscript because it is deemed to be irrelevant for the results. All patients were Caucasian.
Population characteristics	All 4 patients suffered from refractory epilepsy.
Recruitment	We obtained invasive intracranial recordings from four patients with refractory epilepsy treated at UZ Leuven (University Hospital Leuven). These patients were identified by the epileptologist as surgical candidates and underwent intracranial EEG monitoring to localize the epileptic onset zone. At our institution, all patients requiring subdural grid placement for epilepsy monitoring are asked to participate in the study and asked if the consent to the placement of a micro-electrode array. The research protocol was thoroughly discussed with each patient during a preoperative consultation held at least 6 weeks before surgery. Patients were informed about the additional risk associated with micro-electrode array placement, including infection and hemorrhage. Importantly, no additional incision were made for the purpose of this study. Written informed consent was obtained the evening prior to the surgery.
Ethics oversight	Ethische Commissie onderzoek KULeuven/UZLeuven

Note that full information on the approval of the study protocol must also be provided in the manuscript.

Field-specific reporting

Please select the one below that is the best fit for your research. If you are not sure, read the appropriate sections before making your selection.

☒ Life sciences ☐ Behavioural & social sciences ☐ Ecological, evolutionary & environmental sciences

For a reference copy of the document with all sections, see nature.com/documents/nr-reporting-summary-flat.pdf

Life sciences study design

All studies must disclose on these points even when the disclosure is negative.

Sample size	The study included four subjects. The number of patients was entirely dependent on the number of patients requiring intracranial EEG for epilepsy monitoring and was further constrained by the specific electrode placement. For instance, patients requiring only frontal electrodes could not get included in this study.
Data exclusions	In the binocular rivalry experiment, patient 1 misunderstood task instructions and failed to appropriately differentiate between the two images. As a results, it was not possible to analyze perception-related responsed in this patient. However, the non-perception related activity was unaffected and therefore included in this study.

Replication	We tried to replicate the results from each experiment in all four patients. The majority of results could be replicated across arrays.<br>For backward masking, the main findings (obtained in array 2, 4 an 5) could not be replicated in array 3 (because of poor data quality on the day of recording) and array 1 (attributed to that array 1 was positioned in a different anatomical subsection of LO).<br>For flash suppression, the main findings were replicated in array 2,3 and 4, but not in array 1 (not recorded) and array 5. The difference in array 5 is attributed to that neurons did not sustain an elevated spiking rate for an entire second of stimulus presentation (in contrast to some neurons in array 2, 3 and 4).<br>For binocular rivalry, the decoder signaled the identity of the upcoming percept approximately 1500 ms before the button press in array 2, 3 and 4, but not array 5. However, the decoder assigned a higher probability to the upcoming perceived image during the 1500 ms preceding the perceptual report in all arrays. The experiment was not performed in array 1.
Randomization	There was no randomization.
Blinding	There was no blinding.

Reporting for specific materials, systems and methods

We require information from authors about some types of materials, experimental systems and methods used in many studies. Here, indicate whether each material, system or method listed is relevant to your study. If you are not sure if a list item applies to your research, read the appropriate section before selecting a response.

Materials & experimental systems

n/a	Involved in the study
☒	☐ Antibodies
☒	☐ Eukaryotic cell lines
☒	☐ Palaeontology and archaeology
☒	☐ Animals and other organisms
☐	☒ Clinical data
☒	☐ Dual use research of concern
☒	☐ Plants

Methods

n/a	Involved in the study
☒	☐ ChIP-seq
☒	☐ Flow cytometry
☒	☐ MRI-based neuroimaging

Clinical data

Policy information about clinical studies

All manuscripts should comply with the ICMJE guidelines for publication of clinical research and a completed CONSORT checklist must be included with all submissions.

Clinical trial registration	We obtained ethical approval (study number s53126) for conducting semi-chronic microelectrode recordings using the Utah array in patients undergoing invasive epilepsy monitoring.
Study protocol	The full study protocol has been delivered to the editor.
Data collection	Data was collected in the University Hospital Leuven on the epilepsy ward. Patients were recruited between february 2021 and march 2022.
Outcomes	We did not have clinical outcome measures.

Plants

Seed stocks	N/A
Novel plant genotypes	N/A
Authentication	N/A
